# Alternative polyadenylation transcriptome-wide association study identifies APA-linked susceptibility genes in brain disorders

**DOI:** 10.1038/s41467-023-36311-8

**Published:** 2023-02-03

**Authors:** Ya Cui, Frederick J. Arnold, Fanglue Peng, Dan Wang, Jason Sheng Li, Sebastian Michels, Eric J. Wagner, Albert R. La Spada, Wei Li

**Affiliations:** 1 Division of Computational Biomedicine, Department of Biological Chemistry, School of Medicine, University of California, Irvine, Irvine, CA 92697 USA; 2 Departments of Pathology & Laboratory Medicine, Neurology, and Biological Chemistry, School of Medicine, and the UCI Institute for Neurotherapeutics, University of California Irvine, Irvine, CA 92697 USA; 3 Department of Molecular and Cellular Biology, University Baylor College of Medicine, Houston, TX 77030 USA; 4 Department of Medicine, Division of Cardiology, University of California, Los Angeles, Los Angeles, CA 90095 USA; 5 School of Medicine and Dentistry, University of Rochester Medical Center, Rochester, NY 14642 USA

**Keywords:** Computational neuroscience, Gene regulation

## Abstract

Alternative polyadenylation (APA) plays an essential role in brain development; however, current transcriptome-wide association studies (TWAS) largely overlook APA in nominating susceptibility genes. Here, we performed a 3′ untranslated region (3′UTR) APA TWAS (3′aTWAS) for 11 brain disorders by combining their genome-wide association studies data with 17,300 RNA-seq samples across 2,937 individuals. We identified 354 3′aTWAS-significant genes, including known APA-linked risk genes, such as *SNCA* in Parkinson’s disease. Among these 354 genes, ~57% are not significant in traditional expression- and splicing-TWAS studies, since APA may regulate the translation, localization and protein-protein interaction of the target genes independent of mRNA level expression or splicing. Furthermore, we discovered *ATXN3* as a 3′aTWAS-significant gene for amyotrophic lateral sclerosis, and its modulation substantially impacted pathological hallmarks of amyotrophic lateral sclerosis in vitro. Together, 3′aTWAS is a powerful strategy to nominate important APA-linked brain disorder susceptibility genes, most of which are largely overlooked by conventional expression and splicing analyses.

## Abstract (web summary)

Alternative polyadenylation (APA) contributes to the post-transcriptional regulation of most human genes, yet the effects of APA are largely overlooked by conventional transcriptome-wide association studies (TWAS). Here, the authors conduct an APA-TWAS for 11 brain disorders, identifying hundreds of APA-linked disease susceptibility genes.

## Introduction

Alternative polyadenylation (APA) plays an essential role in the post-transcriptional regulation of most human genes^1,2^. By changing the position of polyadenylation (poly(A)), APA can generate transcripts with either shortened or lengthened 3′UTRs that contain different *cis*-regulatory elements, such as binding sites of microRNAs (miRNAs) or RNA-binding proteins (RBPs), leading to altered translation, protein localization, or protein-protein interactions (PPIs) of target genes^3^ independent of mRNA expression level or splicing. The development and differentiation of the nervous system require highly complex post-transcriptional gene regulation networks^4^. APA events resulting in longer 3′UTRs are particularly common during central nervous system (CNS) development^5–7^ and neuronal differentiation^8^. This broad, neural-specific 3’UTR lengthening could mediate miRNA or RBP interactions with 3′UTRs to facilitate cellular localization and targeted translation often observed in neurons. Due to the unique and critical regulatory mechanisms mediated by lengthened 3′UTRs during neurological processes, it is conceivable that single-nucleotide polymorphisms (SNPs) associated 3′UTR length might contribute to brain-related traits and diseases. Indeed, SNPs resulting in aberrant poly(A) site selection have been linked to several neurodegenerative disorders, including the *huntingtin* (*HTT*) gene observed in Huntington’s disease^9^ and *α-synuclein* (*SNCA*) gene in Parkinson’s disease^10^. Moreover, disruption of key APA regulators has been linked to brain-related diseases, such as *PABPN1* in oculopharyngeal muscular dystrophy^11^, *NUDT21* in neuropsychiatric disease^12^, and glioblastoma in our previous study^13^. Together, these observations suggest the relevance of aberrant APA in brain disorders. Nonetheless, the prevalence and functions of SNPs associated with APA for a broad spectrum of brain disorders remains largely unknown.

Genome-wide association studies (GWAS) have identified hundreds of genetic loci associated with an increased risk of developing various brain disorders, including attention deficit hyperactivity disorder (ADHD)^14^, autism spectrum disorder (ASD)^15^, bipolar disorder (BIP)^16^, major depression (MD)^17^, schizophrenia (SCZ)^18^, Parkinson’s disease (PD)^19^, and Alzheimer’s disease (AD)^20^. However, identifying the susceptibility genes responsible for these loci remains a significant challenge, as GWAS data do not reveal how a given SNP affects gene regulation and, ultimately, cellular function. Expression quantitative trait locus (eQTL) analysis has been used recently to identify associations between risk genotypes and gene expression, and several studies have successfully identified putative susceptibility genes related to GWAS risk loci for multiple traits and diseases^21–24^, including psychiatric disorders^25–27^. Nevertheless, despite substantial efforts to identify eQTLs, a large proportion of GWAS SNPs remain unexplained^28,29^. Transcriptome-wide association studies (TWAS) have recently been proposed as the principal method for nominating putative genes associated with disease risk by imputing gene expression levels in large cohorts of individuals^30–34^. In this approach, cohorts with expression and genotype data (e.g., the Genotype-Tissue Expression Project [GTEx]) are used to train gene expression prediction models based on *cis*-SNPs (i.e., within 1 Mbp of the gene). These models are then used to predict gene expression in the GWAS cohorts without directly measuring expression levels. Finally, statistical associations are estimated between predicted gene expression and traits in GWAS cohorts. Compared to GWAS, TWAS have a lower multiple-testing burden and more power to nominate susceptibility genes by aggregating multiple *cis*-SNPs into a single predicted expression value^32,35^. However, these eQTL and TWAS studies do not consider the effects of APA, and thus largely overlook GWAS SNPs associated with post-transcriptional regulation mediated by 3′UTR length changes. Our recent work identified ~0.4 million common genetic variants associated with APA (3′aQTLs) using Genotype-Tissue Expression (GTEx) version 7^36^, which could explain approximately 16.1% of GWAS variants associated with 23 brain-unrelated human traits/diseases^37^. These results underscore the importance of fully characterizing 3′aQTLs and APA-linked susceptibility genes in brain disorders.

In this study, we collect the most comprehensive genome-wide genotype-gene expression datasets to perform 3′aQTL mapping based on 17,300 RNA-seq samples from 2937 individuals, including dorsolateral prefrontal cortex (DLPFC) tissues from 579 individuals in the ROS/MAP cohort, DLPFC tissues from 1,520 individuals in the PsychENCODE cohort, and 838 individuals from GTEx v8 cohort (13 brain tissues and 36 non-brain tissues). The percentage of distal poly(A) site usage index (PDUI) is used as a molecular phenotype to construct a publicly available 3′aTWAS model hub (https://wlcb.oit.uci.edu/3aTWAS) for identifying APA-linked susceptibility genes associated with human diseases or traits. This allows us to conduct the transcriptome-wide association study of 3′UTR usage (3′aTWAS) for 11 brain disorders and we identify 354 APA-linked disease susceptibility genes. Our 3′aTWAS results confirm two previously validated APA risk genes (*SNCA*^10^ and *DDHD2*^38^) and identify many other APA-linked susceptibility genes, approximately 57% of which are overlooked by conventional expression and splicing TWAS, such as *ZNF592* in BIP and SCZ. Furthermore, in the experimental validation of 3′aTWAS-identified genes, we find that modulation of ataxin-3 (*ATXN3*), a 3′aTWAS susceptibility gene for amyotrophic lateral sclerosis (ALS), substantially impacts pathological hallmarks of ALS in vitro. Furthermore, global analyses highlight the convergence of known and previously unknown susceptibility genes in PPI networks and pathways related to brain disorders, including autophagy and membrane trafficking. Taken together, our results present 3′aTWAS as a powerful tool for identifying APA-linked susceptibility genes in brain disorders.

## Results

### 3′aQTLs explain a large portion of brain disorder heritability

**Fig. 1:**
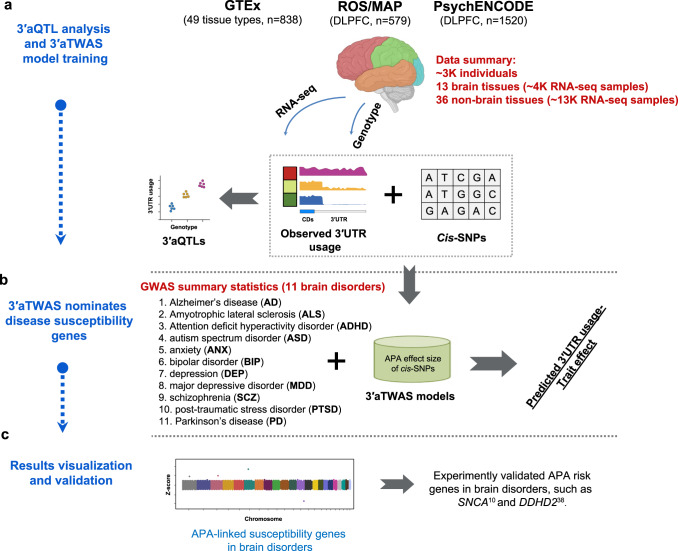
Overview of this study. **a** RNA-seq and matched genotype data were collected from the GTEx, ROS/MAP, and PsychENCODE cohorts as reference panels. We then performed 3′aQTL analysis and built 3′aTWAS models to predict the APA usage of target genes with *cis*-SNPs in the reference panels. **b** We performed 3′aTWAS analysis to nominate susceptibility genes in brain disorders using GWAS summary statistics and 3′aTWAS models in each reference panel. **c** APA-linked susceptibility genes in brain disorders identified by 3′aTWAS, which confirmed two previously validated risk APA genes (*SNCA* and *DDHD2*). This schematic was created with BioRender.

**Fig. 2:**
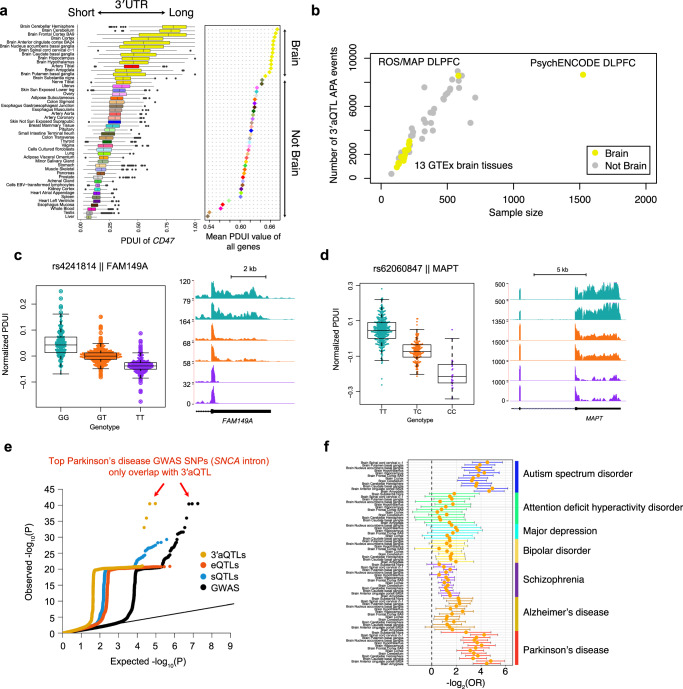
3′aQTLs explain a large portion of brain disorder heritability. **a** PDUI values of 49 GTEx tissues show that transcripts expressed in the brain tissues have longer 3′UTRs than non-brain tissues. The left panel is the PDUI of an example gene *CD47* (*n* = 15,201 RNA-seq samples). The right panel shows the mean PDUI values for all genes. A higher PDUI value corresponds with longer 3′UTR usage. The color of each tissue corresponds with those used in the GTEx cohort. The center horizontal lines within the plot represent the median values and the boxes are bounded by the 25th and 75th percentile. The whiskers extend to the maximum and minimum values within 1.5 times of the interquartile range. **b** The number of 3′aQTL APA events highly correlates with the sample size in each tissue. Each dot indicates a tissue type. Yellow and gray dots indicate brain and non-brain tissue types, respectively. **c** Example of a SNP (rs4241814) that is strongly associated with *FAM149A* 3′UTR usage in the brain. Left panel: Distribution of the normalized PDUI values for each genotype. Each dot in the box plot represents the normalized PDUI value for one particular sample in the ROS/MAP cohort (*n* = 579 biologically independent samples). The center horizontal lines within the plot represent the median values and the boxes are bounded by the 25th and 75th percentile. The whiskers extend to the maximum and minimum values within 1.5 times of the interquartile range. Right panel: RNA-seq coverage tracks for the *FAM149A* 3′UTR. The bottom track shows the RefSeq gene structure. **d** Similar to (**c**) but for *MAPT* in the PsychENCODE cohort (*n* = 1520 biologically independent samples). The center horizontal lines within the plot represent the median values and the boxes are bounded by the 25th and 75th percentile. The whiskers extend to the maximum and minimum values within 1.5 times of the interquartile range. **e** Example of Parkinson’s disease quantile–quantile plot (QQ plot) showing the nominal *P*-values of brain disorder GWAS SNPs, which were binarily annotated by 3′aQTLs (yellow), sQTLs (blue), and eQTLs (light orange) with nominal *P*-value < 10^−5^. Each dot represents a GWAS SNP. All Parkinson’s disease GWAS nominal *P*-values are also shown as controls (black). **f** Enrichment of 3′aQTLs in seven brain disorder GWAS SNPs (nominal *P*-value < 10^−5^) across GTEx brain tissues. 3′aQTLs are calculated based on 2181 RNA-seq samples of brain tissues from the GTEx cohort. Data are presented as mean values ± SEM. OR odds ratio.

First, we performed 3′aQTL analysis, as described in our previous study^36^, to identify local genetic effects associated with variations in 3′UTR usage in 17,300 genotype-matched RNA-seq samples from the ROS/MAP, PsychENCODE, and GTEx v8 cohorts (Fig. 1a). To the best of our knowledge, this study represents the largest 3′aQTL study of the human brain. We used our previously described DaPars2^39,40^ algorithm to identify APA events. Briefly, the DaPars2 framework calculates a percentage of distal poly(A) site usage index (PDUI), which can identify 3′UTR lengthening or shortening events by joint analyses of all RNA-seq samples. All PDUI values were normalized after corrections for known covariates, including sex, sequencing platform, RNA integrity number (RIN), post-mortem interval, and population structure, as well as hidden covariates inferred by probabilistic estimation of expression residual (PEER) factors^41^. Consistent with previous studies^5,6^, cross-tissue PDUI values reveal that the brain harbors the longest 3′UTRs among all tissue types (Fig. 2a), suggesting an intriguing possibility that APA might play a particularly important role in normal brain function and implying that its dysfunction could play a central role in brain disorders. Indeed, we found that many GWAS lead SNPs in brain disorders are located near the 3′UTR of target genes, such as *ARL17B* (Supplementary Fig. 1). We then used Matrix eQTL^42^ to identify *cis* SNPs (within 1Mbp of a 3′UTR) associated with differential 3′UTR usage (3′aQTLs), as described previously^37^. Using a false-discovery rate (FDR) threshold of 5%, we identified in total ~12.9 M 3′aQTLs for 16,301 genes in three cohorts (Fig. 2b). Many of these 3′aQTLs were associated with important brain disease-related genes, such as *FAM149A* (Fig. 2c) for Glass Syndrome^43^ and *MAPT* (encoding *tau* protein) for PD and AD (Fig. 2d). Our current 3′aQTL results from 2937 individuals greatly extended our previous 3′aQTL studies (467 individuals^37^ and 838 individuals^44^), particularly for brain tissues (representing a ~3.5-fold increase in individuals).

We next explored the extent to which 3′aQTLs could explain brain disorder heritability. First, we used quantile-quantile plots (QQ-plots) to visualize the *P*-values of brain disorder GWAS SNPs, which were binarily annotated according to 3′aQTLs from GTEx Brain Cortex tissue with *P* < 1 × 10^−5^ (Fig. 2e and Supplementary Fig. 2). For comparison, we also included eQTLs and sQTLs from the same dataset in the same plot (Fig. 2e). All three QTL types showed significant overlap with GWAS risk SNPs of brain disorders, such as PD (Fig. 2e). Interestingly, the top PD GWAS SNPs, located near the α-synuclein gene (*SNCA*), overlapped only with 3′aQTLs but not with eQTLs and sQTLs (Fig. 2e). *SNCA* is known to be a major PD risk gene, and an increase in *SNCA* gene expression is known to cause parkinsonism in affected families. Our results showed that the lead PD GWAS SNP rs356203 has a very significant 3′aQTL signal but no eQTL signal, which is consistent with the previous finding that APA mediates the GWAS risk associated with this SNP in *SNCA*^10^. Furthermore, applying previously reported strategies^45^, we used GARFIELD^46^ to assess 3′aQTL enrichment in seven brain disorder GWAS SNPs. GARFIELD is a functional enrichment tool that controls for linkage disequilibrium (LD), allele frequency, and distance to genes. We found a 2–5 (log_2_) fold enrichment of 3′aQTLs in several brain disorders in various brain tissues (Fig. 2f). Together, these results demonstrate that APA represents an important molecular phenotype capable of explaining a large portion of genetic susceptibility associated with brain disorders.

### Development of 3′aTWAS prediction models

**Fig. 3:**
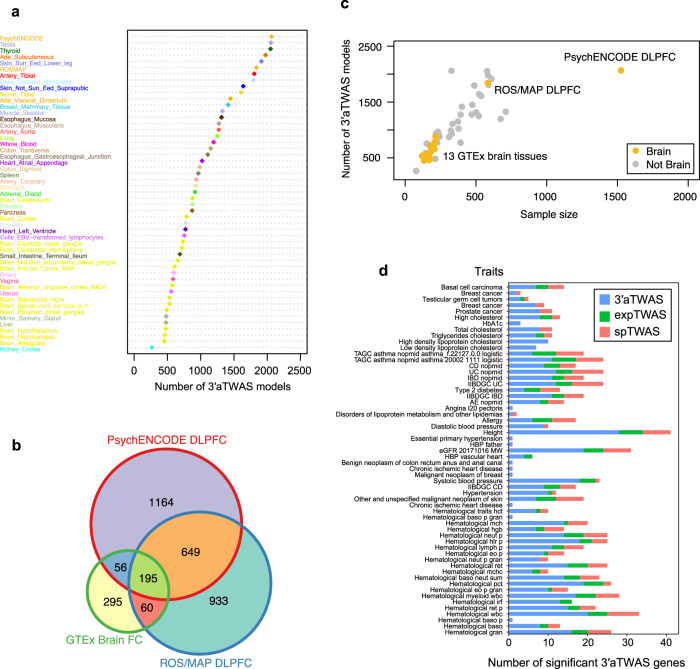
3′aTWAS hub across 13 brain tissues and 36 non-brain tissues from ROS/MAP, PsychENCODE, and GTEx cohorts. **a** Number of 3′aTWAS models across each tissue from ROS/MAP, PsychENCODE, and GTEx cohorts. DLPFC, dorsolateral prefrontal cortex. **b** Venn diagram showing the overlap between the number of 3′aTWAS models in ROS/MAP DLPFC, PsychENCODE DLPFC, and GTEx Brain frontal cortex (FC) tissues. **c** The number of 3′aTWAS models is highly correlated with the sample size in each tissue. Each dot indicates a tissue type. Yellow and gray dots indicate brain and non-brain tissue types, respectively. **d** A majority of human diseases of 3′aTWAS genes are not expression TWAS or splicing TWAS genes. 3′aTWAS specific genes are shown in blue. Overlap between 3′aTWAS and expression TWAS genes is shown in green. Overlap between 3′aTWAS and splicing TWAS genes is shown in red.

To systematically identify APA-linked susceptibility genes associated with human brain disorders, we repurposed the traditional TWAS methodology (FUSION software^32^) to examine the association between GWAS summary statistics and 3′UTR usage (termed 3′aTWAS) instead of gene expression (Fig. 1b, c). Briefly, for each dataset, we used a mixed-linear model to estimate the heritability of 3′UTR usage (normalized PDUI values after corrections for covariates) explained by *cis-*SNPs proximal to the 3’UTR of each gene in a reference panel (cohorts including matched RNA-seq and genotype data). Only genes with significant (*P*-value < 0.05) heritability estimates (*cis*-h^2^) were included in the following analyses. For each FUSION-trained model, including BLUP, LASSO, and Elastic Net, we used cross-validation to choose the model with the best 3′aTWAS prediction accuracy for each gene. We obtained a total of 52,829 tissue-specific 3′aTWAS PDUI prediction models in the ROS/MAP, PsychENCODE, and GTEx reference panels (Fig. 3a), spanning 10,508 unique APA events in 7809 unique genes. Of note, the same APA event could have multiple PDUI prediction models for different tissues. We also found that 51.53% (5415/10,508) of 3′aTWAS models are in at least two reference panels (Fig. 3b and Supplementary Fig. 3), with an average of 1035 3′aTWAS models per panel.

The number of 3′aTWAS prediction models was highly correlated with the sample sizes of the reference panels (Fig. 3c). Comparing with Frontal Cortex BA9 in the GTEx v8 panel (175 individuals), the two panels with larger sample sizes, PsychENCODE (1520 individuals) and ROS/MAP (579 individuals), had 3.41-fold (2064/606) and 3.03-fold (1837/606) more 3′aTWAS prediction models, respectively (Fig. 3c). The strong correlation between the number of 3′aTWAS prediction models and sample size (Pearson correlation *P*-value < 7.76e−12, *r* = 0.79) suggests that more APA genes will continue to be predicted as additional RNA-seq datasets become available (Fig. 3c). Given the current datasets, the average in-sample prediction accuracies (measured by heritability normalized *R*^2^, *R*^2^/*cis-h*^2^) of 3′aTWAS models were 80.45%, 77.08%, and 67.31% for the ROS/MAP, PsychENCODE, and GTEx Frontal Cortex BA9 panels, respectively (Supplementary Fig. 4), which were similar to the previous expression and splicing TWAS models. These results indicated that similar to expression and splicing TWAS models, most cis-regulated 3’UTR usage (PDUI value) information is captured by *cis*-SNPs. Finally, we tested 3′aTWAS individual tissue models for 31 human traits (18 brain-related and 13 brain-unrelated). Our results showed that the majority of 3′aTWAS-identified genes are not expression TWAS or splicing TWAS genes (Fig. 3d). In summary, we have developed highly comprehensive 3′aTWAS prediction models, which uncovered susceptibility genes that were not previously captured by expression or splicing TWAS.

### 3′aTWAS for 11 brain disorders

**Fig. 4:**
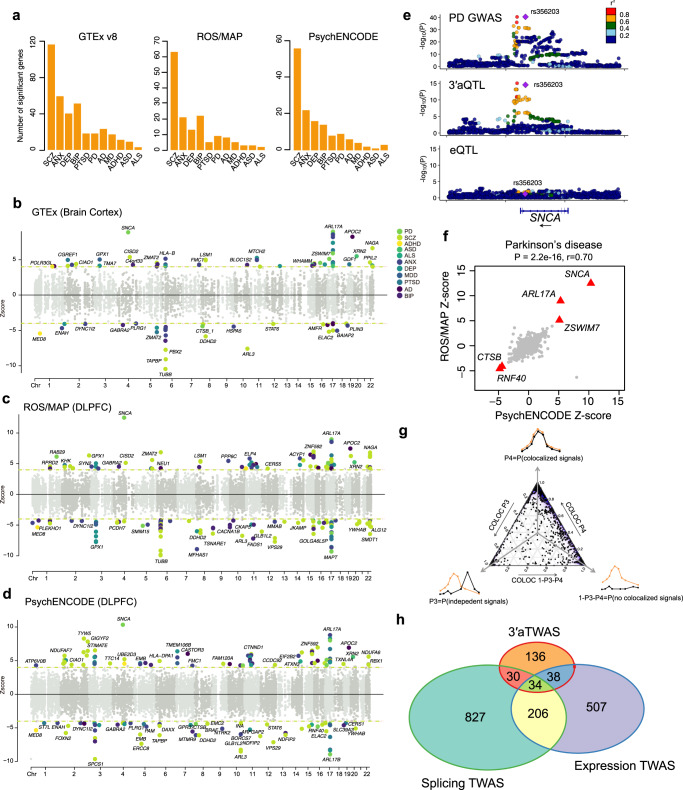
3′aTWAS for 11 brain disorders. **a** Bar plots show the number of 3′aTWAS significant genes (FDR < 0.05) for 11 brain disorders in 13 GTEx-derived brain tissues, ROS/MAP DLPFC, and PsychENCODE DLPFC. **b**–**d** Manhattan plots of 3′aTWAS results in 11 brain disorders using prediction models from GTEx Brain Cortex (**b**), ROS/MAP DLPFC (**c**), and PsychENCODE DLPFC (**d**). Each point represents the *Z*-score of a single 3′aTWAS association. Colored points represent significant associations with brain disorders at FDR < 0.05, with each of the 11 colors representing 1 of 11 different brain disorders. **e** Aligned Manhattan plots of Parkinson’s disease GWAS, 3′aQTLs, and eQTLs at the *SNCA* locus. SNPs are colored by LD (*r*^2^). **f** Parkinson’s disease was used as an example to assess whether similar results were observed from different 3′aTWAS prediction models built from an independent reference panel. Parkinson’s disease 3′aTWAS *Z*-scores in ROS/MAP and PsychENCODE are highly correlated (two-tailed Pearson correlation *P*-value = 2.2e−16, *r* = 0.70). Red triangles represent replicate genes in Parkinson’s disease which are 3′aTWAS significant and have consistent directions when using ROS/MAP and PsychENCODE as reference panels. **g** A ternary plot represents the colocalization probabilities for 3′aTWAS significant associations. **h** Venn diagram shows the overlap of 3′aTWAS significant genes (FDR < 0.05) for 11 brain disorders with expression and splicing TWAS.

We applied the brain tissue-trained 3′aTWAS models to the GWAS summary statistics of 11 brain disorders, including amyotrophic lateral sclerosis (ALS)^47^, attention deficit hyperactivity disorder (ADHD)^14^, autism spectrum disorder (ASD)^15^, anxiety (ANX)^48^, bipolar disorder (BIP)^16^, depression (DEP)^48^, major depressive disorder (MDD)^17^, schizophrenia (SCZ)^18^, post-traumatic stress disorder (PTSD)^49^, Parkinson’s disease (PD)^50^ and Alzheimer’s disease (AD)^20^ (Fig. 1b-c and Supplementary Table 1). In total, we identified 1,393 significant associations between predicted PDUI values and GWAS phenotypes (FDR < 0.05), representing 449 transcripts of 354 APA-linked disease susceptibility genes (Fig. 4a-d and Supplementary Data 1). Among these 354 susceptibility genes, 121 were identified in more than one reference panel. The largest number of APA transcripts were associated with SCZ in all three panels (78 in ROS/MAP, 62 in PsychENCODE, and 141 in 13 GTEx brain tissues; Fig. 4a). We also found susceptibility genes that were shared across multiple brain disorders (Supplementary Fig. 5). For example, 19 genes were common to both SCZ and BIP, including *BORCS7*, which is a known molecular risk factor for SCZ^51^. Next, we considered the LD and co-regulation caveats in TWAS analysis^52^ and used a Bayesian fine-mapping method FOCUS^52^ to prioritize putatively causal 3′aTWAS genes. 151 transcripts were prioritized as putatively causal candidates with a posterior inclusion probability (PIP) > 0.9 (Supplementary Data 2).

To validate APA-linked susceptibility genes identified by 3′aTWAS, we performed the following analyses. First, as a positive control, we confirmed that the 3′aTWAS models could identify previously reported APA-linked disease susceptibility genes, e.g., *SNCA*^10^ and *DDHD2*^38^, with risk SNPs within their 3′UTRs. In PD, *SNCA* was the most significant gene identified in multiple reference panels, including ROS/MAP (*P*-value = 7.80e−36), PsychENCODE (*P*-value = 5.08e−25), GTEx Brain Cerebellar Hemisphere (*P*-value = 2.23e−10), GTEx Brain Cerebellum (*P*-value = 3.87e−20), GTEx Brain Cortex (*P*-value = 6.47e−19), and GTEx Brain Frontal Cortex BA9 (*P*-value = 4.10e−7). In agreement with previous studies^10^, *SNCA* had a positive *Z*-score, indicating that long 3′UTR usage in *SNCA* increases PD risk. The leading PD GWAS SNP near *SNCA* (<1 Mbp) is in high LD (*R*^2^ = 0.99) with the lead 3′aQTL SNP rs356165 (*P*-value = 3.07e−15) of *SNCA*, but this leading GWAS SNP is less strongly associated with eQTLs and sQTLs of *SNCA* in GTEx Brain Cortex (*P*-value > 0.00001; Fig. 4e and Supplementary Fig. 6). This suggests that 3′UTR usage, rather than mRNA expression or splicing of the *SNCA* transcript, mediates risk in PD. Additionally, our 3′aTWAS models identified *DDHD2* as a significant SCZ susceptibility gene across ROS/MAP, PsychENCODE, and 13 GTEx-derived tissue types, consistent with previous reports^38^. Specifically, a negative Z-score of *DDHD2* in these reference panels indicates that short 3′UTR usage of *DDHD2* increases SCZ risk (Fig. 4b–d).

We further used PD as an example to assess the robustness of the associations between GWAS phenotypes and predicted PDUIs from 3′aTWAS models trained with different reference panels. We found that the PD 3′aTWAS association *Z*-scores based on ROS/MAP and PsychENCODE cohorts were highly correlated with one another (Pearson correlation *P*-value < 2.2e−16, *r* = 0.70) (Fig. 4f). Furthermore, five out of nine significantly associated genes (*SNCA*, *ARL17A*, *ZSWIM7*, *NCOR1*, and *RNF40*) from the PD ROS/MAP cohort were replicated in the PsychENCODE cohort at an FDR < 0.05, including four genes with consistent effect directions. These replication results demonstrate that 3′aTWAS risk genes are highly robust regardless of which reference panel is used to train the models.

Interestingly, we also found that many significant 3′aTWAS genes are far from significant GWAS loci—all significant (*P*-value < 10^−8^) GWAS loci are at least 1 Mbp away from the 3′aTWAS gene. These risk genes were identified because 3′aTWAS has more power to nominate susceptibility genes through aggregating multiple *cis*-SNPs into a single predicted PDUI value^32,35^. For example, the best proximal GWAS SNP within 1 Mbp of a 3′aTWAS gene *RABEP1* in SCZ has a non-significant *P*-value of 1.77 × 10^−6^ (Supplementary Fig. 7). Similarly, the best proximal GWAS SNP within 1Mbp of a 3′aTWAS gene *CHDH* in BIP has a non-significant *P*-value of 6.40 × 10^−7^ (Supplementary Fig. 8).

We also conducted colocalization analysis using coloc software^53^ to identify APA genes that share 3′aQTLs with GWAS SNPs. Briefly, coloc calculates five posterior probabilities (PP0–PP4), among which PP4 represents the probability that GWAS signals and 3′aQTLs share causal SNPs. Here, we considered PP4 ≥ 0.5 as significant colocalization. In total, 55.42% (772/1,393) of 3′aTWAS associations, 56.12% (252/449) of transcripts, and 54.80% (194/354) of genes have high colocalization probabilities (Fig. 4g and Supplementary Data 1). Of note, compared with the TWAS method, the colocalization method has limited power for the identification of multiple independent causal variants per gene. Furthermore, we used SuSiE^54^ to fine-map 3′aQTLs for all significant 3′aTWAS genes. We found that many 3′aTWAS genes, such as *STAT6*, *PPIL2*, and *ELAC2* (Supplementary Fig. 9), had at least one SuSiE-identified potentially causal SNP with high posterior inclusion probability (PIP > 0.7). A table of all SNPs with PIP > 0.7 was provided in Supplementary Data 3. Finally, we compared 3′aTWAS with both classical expression and splicing TWAS. In total, a large portion (57.14%, 136/238) of 3′aTWAS genes were not identified by either expression or splicing TWAS (Fig. 4h). The 3′aTWAS genes identified for 11 brain disorders are publicly available (https://wlcb.oit.uci.edu/3aTWAS), which we hope will serve as a useful resource for the neuroscience research community.

### 3′aTWAS identifies important APA-linked susceptibility genes in brain disorders

**Fig. 5:**
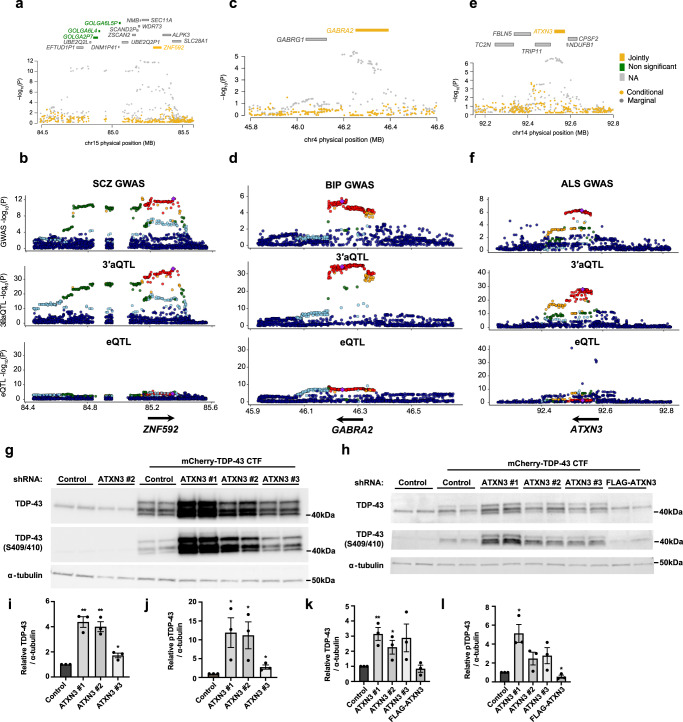
3′aTWAS identifies new APA-linked susceptibility genes in brain disorders. **a** Regional association plot. SCZ GWAS signal at the *ZNF592* locus (gray) and GWAS signal after removing the effects of *ZNF592* 3′UTR usage (yellow). This analysis shows that the association is largely explained by *ZNF592* 3′UTR usage. **b** Aligned Manhattan plots of SCZ GWAS, 3′aQTLs, and eQTLs at the *ZNF592* locus. SNPs are colored by LD (*r*^2^). **c** Similar to (**a**) for the *GABRA2* locus in BIP. **d** Similar to (**b**) for the *GABRA2* locus in BIP. **e** Similar to (**a**) for the *ATXN3* locus in ALS. **f** Similar to (**b**) for the *ATXN3* locus in ALS. **g-h** Western analysis of HEK293T cells (**g**) and SH-SY5Y cells (**h**) transfected with mCherry-tagged TDP-43 CTF (aa 208–414) and shRNAs for 48 h. Note: Multiple bands reflect distinct cleavage products. TDP-43 was detected using an antibody that recognizes a C-terminal epitope. **i** Quantification of total TDP-43 in HEK293T cells transfected with ATXN3 shRNA #1 (*p* = 0.0013), #2 (*p* = 0.0016), or #3 (*p* = 0.0182) relative to shRNA control. **j** Quantification of pTDP-43 in HEK293T cells transfected with ATXN3 shRNA #1 (*p* = 0.0483), #2 (*p* = 0.0460), or #3 (*p* = 0.0351) relative to shRNA control. **k** Quantification of total TDP-43 in SH-SY5Y cells transfected with ATXN3 shRNA #1 (*p* = 0.0073), #2 (0.0496), #3 (0.1098), or FLAG-ATXN3 (*p* = 0.5485) relative to shRNA control. **l** Quantification of pTDP-43 in SH-SY5Y cells transfected with ATXN3 shRNA #1 (*p* = 0.0121), #2 (*p* = 0.0961), #3 (*p* = 0.01050), or FLAG-ATXN3 (*p* = 0.0488) relative to shRNA control. Each experiment was repeated *n* = 3 times. Data are presented as mean values ± SEM. Statistical significance was determined by an unpaired two-tailed *t*-test between each condition and the shRNA control. * represents *p* < 0.05. ** represents *p* < 0.01.

In addition to the previously reported APA-linked susceptibility genes, *SNCA* and *DDHD2*, our 3′aTWAS analysis identified previously unknown APA-linked susceptibility genes (352 of 354) in a wide range of brain disorders, most of which were overlooked by conventional expression TWAS and splicing TWAS analyses. This indicates that GWAS risk loci associated with these newly described susceptibility genes can only be explained by APA, independent of gene expression and splicing changes. For example, the 3′aTWAS gene *ZNF592* (both identified in SCZ and BIP), which was not identified by expression or splicing TWAS, has been reported to play a key role in cerebellar development^55^. Mutations in *ZNF592* can cause familial mental retardation (CAMOS syndrome)^55^. After adjusting for the predicted 3′UTR usage of *the ZNF592* gene, the SCZ GWAS signal substantially decreases (Fig. 5a), indicating that the 3′aTWAS association of *ZNF592* almost entirely explains the SCZ GWAS signal in this region. The *ZNF592* 3′aQTL also exhibits high colocalization probability with the SCZ GWAS signal (PP4 = 0.983), but not with *ZNF592* eQTLs or sQTLs (Fig. 5b). Our data suggest the importance of *ZNF592* APA regulation in SCZ. Two similar examples (*HP1BP3* and *SYN2*) are shown in Supplementary Fig. 10.

Furthermore, our 3′aTWAS results revealed that many known brain disorder GWAS risk loci-associated genes are heavily regulated by APA, including some that were also identified by expression TWAS or splicing TWAS. Of note, many of these overlapping genes have a stronger 3′aQTL signal than eQTL or sQTL signal, indicating that dysregulation of APA in these genes plays a prominent role in related brain disorders. For example, 3′aTWAS identified *GABRA2* (encoding the GABAA receptor) as an important neuronal inhibition regulator implicated in many brain disorders^56,57^. We found strong 3′aQTLs but relatively weak eQTLs for *GABRA2* in ROS/MAP (lead eQTL: *P* = 6.47e−10, lead 3′aQTL: *P* = 1.91e−38). Similar to *ZNF592*, the 3′aTWAS association of *GABRA2* almost entirely explains the BIP GWAS signal in this region (Fig. 5c). The *GABRA2* 3′aQTLs also show high colocalization probability with the BIP GWAS signal (PP4 = 0.955), but not with the *GABRA2* eQTL (Fig. 5d). Similarly, *CTSB*, which is essential in α-synuclein lysosomal degradation and Parkinson’s disease^58^, has more significant 3′aQTLs than eQTLs in the GTEx Brain Cortex cohort (lead eQTL: *P* = 6.28e−06, lead 3′aQTL: *P* = 4.48e−19) (Supplementary Fig. 11a); *DYNC1I2* has more significant 3′aQTLs than eQTLs in the GTEx Brain Cerebellum cohort (lead eQTL: *P* = 9.50e−07, lead 3′aQTL: *P* = 2.11e−44) (Supplementary Fig. 11b).

Overall, our 3′aTWAS results provide a valuable resource of susceptibility genes for a wide array of brain disorders, which warrant experimental validation. As an example, we focused on *ATXN3* in ALS, as *ATNX3* 3′aQTLs showed the highest colocalization probability with the ALS GWAS signal (PP4 = 0.969, Supplementary Table 2), but not with *ATNX3* eQTLs (Fig. 5e, f). To this end, we examined the potential role of the 3′aTWAS-identified ALS susceptibility gene *ATXN3* on key ALS pathological hallmarks in vitro. Ubiquitin-positive, cytoplasmic inclusions containing hyperphosphorylated TDP-43 C-terminal fragments (CTFs) are observed in post-mortem tissue of approximately 97% of ALS patients^59,60^. Given that ATXN3 is a deubiquitinase that regulates cellular proteostasis, we investigated whether modulating the protein levels of ATXN3 impacts TDP-43 aggregation. While endogenous, full-length TDP-43 does not normally aggregate in cells, this pathology can be readily induced by transient overexpression of aggregation-prone TDP-43 CTFs^61^. Indeed, we found that small hairpin RNA (shRNA)-mediated *ATXN3* knockdown (Supplementary Fig. 12) results in the substantial accumulation and hyperphosphorylation of TDP-43 CTFs at S409/S410 in HEK293T cells (Fig. 5g, i, j) and SH-SY5Y neuroblastoma cells (Fig. 5h–l). FLAG-ATXN3 overexpression reduced the accumulation and phosphorylation of TDP-43 CTFs in SH-SY5Y cells (Fig. 5h–l), demonstrating that ATXN3 modulation significantly affects key ALS molecular phenotypes in vitro. Given the role of ATXN3 in the ubiquitin–proteasome system, we tested the hypothesis that ATXN3 knockdown disrupts TDP-43 CTF degradation. Indeed, we observed a significant increase in TDP-43 CTF stability upon *ATXN3* knockdown (Supplementary Fig. 13). Future experiments are required to determine the functional significance of *ATXN3* APA on the metabolism of the *ATXN3* transcript and to investigate the potential role of *ATXN3* APA in ALS. In summary, these results indicate the ability of 3′aTWAS to reveal functionally important susceptibility genes associated with brain disorders.

### 3′aTWAS brain disorder genes are enriched in autophagy and membrane trafficking pathways

**Fig. 6:**
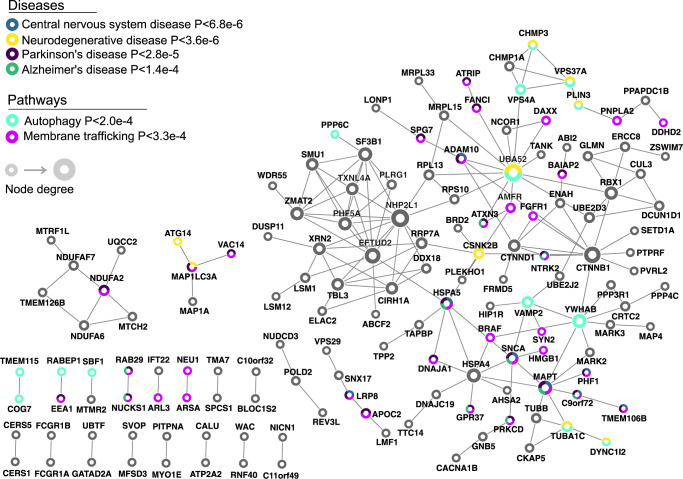
3′aTWAS prioritizes known and previously unknown susceptibility brain disorder genes that are connected in PPI networks and enriched in autophagy and membrane trafficking pathways. 3′aTWAS genes are connected in PPI networks with known brain disorder genes. Pathway enrichment analysis showed that 3′aTWAS genes are enriched in brain disorder related pathways, including autophagy and membrane trafficking pathways. Each node represents one 3′aTWAS gene. Node size represents the node degree.

We used inBio Discover^62^ to evaluate whether known disease-related genes (defined by inBio Discover) and brain disorder susceptibility genes identified by 3′aTWAS converge on similar functional networks or pathways. Our results showed that 3′aTWAS genes were enriched in CNS diseases, neurodegenerative diseases, PD, and AD (Fig. 6). PPI analyses highlight the convergence of known and previously unknown susceptibility genes in PPI networks (Fig. 6). Furthermore, 3′aTWAS genes were also enriched in the autophagy pathway (*P* < 2.0e-4), consistent with previous work demonstrating that autophagy is critical in CNS diseases^63,64^. Autophagy plays a key role in the maintenance of cellular proteostasis by facilitating the degradation of misfolded proteins and protein aggregates—hallmark pathological features of several neurodegenerative disorders. Additionally, as a macromolecular recycling system, autophagy responds to nutrient and energy levels in the cell to maintain metabolic homeostasis^63–65^. We also found enrichment of 3′aTWAS brain disorder genes in the membrane trafficking pathway (*P* < 3.3e−4) (Fig. 6), further highlighting the importance of membrane trafficking in brain disorders^66^. Membrane trafficking is essential for preserving neuronal heath from the soma to distant axons and dendrites, as these microcompartments require continuous protein transport and clearance. Correspondingly, disruption of membrane trafficking has been observed in many CNS disorders^66–68^. Notably, membrane trafficking and autophagy are tightly linked processes, as autophagosomes that initiate in axons and dendrites require trafficking to the soma, where degradative lysosomes are found in neurons^69^. This may suggest that 3′aTWAS genes are broadly enriched in cellular pathways involved in maintaining neuronal proteostasis. In summary, these results suggest that genes identified by 3′aTWAS represent important components of gene regulatory networks in the brain and that APA dysregulation represents an important, understudied mechanism involved in brain disorders.

## Discussion

In this study, we developed 3′aTWAS to systematically identify APA-linked susceptibility genes, and provide evidence that 3′UTR usage associated with common SNPs is an important feature of brain disorder, capable of explaining GWAS risk loci that are not associated with dysregulation of gene expression or splicing. We built 3′aTWAS prediction models in the largest brain-relevant tissue datasets available (ROS/MAP, PsychENCODE, and GTEx Consortia) and used these prediction models to test whether the effects of risk SNPs in brain disorders were mediated by changes in 3′UTR usage. Our 3′aTWAS results not only confirmed previously reported APA risk genes (*SNCA* and *DDHD2*) but also identified previously unknown APA-linked susceptibilities genes, such as *ZNF592* and *ATXN3*. Further analysis revealed that 3′aTWAS-identified genes are not a random set of genes but are enriched in previously reported pathways related to brain disorders. To the best of our knowledge, this study represents the most comprehensive effort to evaluate the association of genetically predicted 3′UTR usage with complex human diseases.

We demonstrate that 3′aTWAS serves as a powerful tool for identifying APA-linked susceptibility genes. Further experimental study of these genes will not only increase our understanding of the molecular mechanisms underlying various brain disorders but will also highlight candidate genes for therapeutic intervention. In this study, we showed that the 3′aTWAS gene *ATXN3* directly impacts key pathological ALS hallmarks in vitro. *ATXN3* knockdown substantially increased the accumulation and phosphorylation of TDP-43 CTFs, whereas *ATXN3* overexpression reduced these phenotypes in human neuroblastoma cells. ATXN3 is a deubiquitinase with several reported cellular functions, including the regulation of proteasomal degradation of ubiquitinated proteins^70^. ATXN3 physically and functionally interacts with familial ALS-associated proteins, binding and co-aggregating with valosin-containing protein (VCP)^71^ and regulating the recruitment of misfolded superoxide dismutase 1 (SOD1) into aggresomes^72^. CAG-polyglutamine (polyQ) repeat expansions in ATXN3 cause the neurodegenerative disorder spinocerebellar ataxia type 3 (SCA3). SCA3 patients uniformly display cerebellar and brainstem degeneration, and many SCA3 patients develop motor neuronopathy^73^. Furthermore, pathological TDP-43 inclusions have been detected in lower motor neurons in the brainstem and spinal cord of SCA3 patients, which resembles findings in ALS patients^74^. Although intermediate-length CAG-polyQ repeats in the ataxin-2 gene have been linked to ALS, CAG-polyQ repeat length in *ATXN3* has not been associated with ALS risk^75^. As a proof-of-concept, we showed that *ATXN3* modulation affects ALS molecular phenotypes in vitro. We note that this does not provide evidence for a causative role of *ATXN3* APA in ALS, and future studies will be required to investigate the link between *ATXN3* APA and ALS more deeply.

There are several potential reasons that 3′aTWAS is able to identify many genes that are not significant in traditional expression- and splicing-TWAS studies. Mechanistically, APA regulation differs greatly from that of gene expression and splicing. APA is primarily regulated by APA-specific regulators, including the canonical polyadenylation factors (e.g., CFIm25) and various RNA- or DNA-binding proteins^76,77^, but not by transcription factors or splicing factors. Additionally, genetically driven APA change can impact ribosome occupancy and protein expression levels without affecting mRNA expression levels^78^. Furthermore, previous studies showed that 3′aQTLs have distinct molecular features and are largely distinct from other QTLs, such as expression and splicing QTLs^37,78^.

Future studies will be required to identify which SNPs drive changes in APA usage in the APA-linked disease susceptibility genes identified in this study. It remains challenging to identify the causal variant among correlated variants for QTLs due to LD; however, further fine-mapping analyses, including statistical and functional fine-mapping methods^79^, may solve part of the problem. Statistical fine-mapping methods, such as eCAVIAR^80^, FINEMAP^81^, or SuSiE^54^, can prioritize potential causal variants using a variety of methods such as the heuristic, penalized regression, and Bayesian strategies^79^. However, these methods often cannot distinguish causal variants from variants in strong LD. Recently, functional fine-mapping methods, such as the massively parallel reporter assay (MPRA)^82^, have successfully identified putative causal variants for both mRNA expression^82^ and 3′UTR usage^83^. In contrast to other methods, MPRA is able to identify multiple causal variants even in a single tight LD region^82^. After identifying the causal variants for 3′aTWAS genes, CRISPR-based experiments could be used to validate the impact of SNPs on 3′UTR usage and ultimately on disease-related phenotypes. Importantly, there are a growing number of approaches to modulate APA in vitro and in vivo^84,85^, which may eventually be developed into disease-modifying therapies.

Moving forward, there are three main challenges that may prevent the leveraging of 3′aTWAS in elucidating the mechanistic basis of neurodegenerative and neuropsychiatric disorders. First, a large sample size of matched transcriptomic and genomic datasets from different brain regions is required to build more accurate prediction models. Although transcriptomics data from multi-brain regions exist in the GTEx cohort, the sample size is as of date too limited (*n* = 114–209) to build robust and accurate 3’aTWAS models for most genes. The strong correlation between the number of 3′aTWAS prediction models and sample size in this study suggests that limited sample size remains an issue for most brain tissue types and that more APA genes will continue to be predicted as additional RNA-seq datasets become available. Second, most samples in the available transcriptome cohorts (e.g., GTEx, ROS/MAP, and PsychENCODE) were derived from individuals of Caucasian-European ancestry. More diverse transcriptome datasets (e.g., African and Asian racial ancestry) remain necessary to build ancestry-specific 3′aTWAS prediction models. Third, similar to eQTLs or sQTLs, 3′aQTLs are often cell type-specific and condition-specific. Using bulk and steady-state RNA-seq datasets, current 3′aQTLs may not include genetic effects that are only active in disease-relevant cell types or those that can be modified by environmental conditions, such as stimulation or viral infection. Recent studies have suggested that a large fraction of eQTLs strongly associate with genotype in a cell-type- or condition-specific context^86^. Therefore, the construction of cell type-specific and condition-specific 3’aTWAS prediction models under various disease-related cell types and conditions represent a major future direction.

In summary, our 3′aTWAS prediction model serves as a new resource for investigating the effects of genetic variants affecting APA. While we have initially focused on brain disorders in this study, our 3′aTWAS prediction models across 49 human tissues can be readily applied to many other human diseases and traits. 3′aTWAS provides novel insights into previously unexplained GWAS risk loci and implicates APA as a major contributing factor to the pathogenesis of the human disease.

## Methods

### Transcriptomics cohorts in this study

The RNA-seq and matched genotype data in the following cohorts were used in our study: (1) ROS/MAP Consortium: We downloaded RNA-seq and whole genome sequencing (WGS) data of ROS/MAP from the AMP-AD Consortium^87,88^. We included only RNA-seq samples that were generated from post-mortem human DLPFC and excluded RNA-seq samples without matched WGS data. When multiple samples were derived from the same individuals, we selected the samples with the highest RIN. Raw RNA-seq data were generated from post-mortem human DLPFC tissue and mapped to the human genome (hg19/GRCh19) using STAR^89^ as previously described^90^. Genotype VCF files were downloaded from the AD Knowledge Portal. (2) PsychENCODE Consortium: DLPFC RNA-seq data and matched genotype files from PsychENCODE^27,91^ were included in the following analyses. We selected samples with the highest RIN when multiple samples were derived from the same individual. Original RNA-seq data were also mapped to the human genome (hg19/GRCh19) using STAR^89^. Genotype VCF files were downloaded from the PsychENCODE “PEC Capstone Collection”. Variants with low imputation quality (*r*^2^ ≤ 0.7) were removed by PLINK^92^. (3) GTEx Consortium: Raw GTEx RNA-seq and genotype files were obtained from the dbGaP database (phs000424.v8.p2), which includes 17,382 RNA-seq samples across 54 tissues from 948 individuals. We removed tissue types with small sample sizes, including the brain substantia nigra, bladder, cervix endocervix, cervix ectocervix, fallopian tube, and minor salivary gland. Only individuals included in the GTEx analysis freeze were considered. For all three cohorts, variants were filtered to remove variants with minor allele frequency ≤ 0.01, indels, and all variants with ambiguous ref/alt alleles. Five genotype principal components (PCs) were computed to account for ancestry covariates in all subsequent analyses. Sample collection, data generation, and data processing were approved by the institutional review board (IRB) of each cohort. This study was approved by the IRB of the University of California, Irvine (UCI IRB #431). All data derived from human post-mortem samples used in this study comply with all relevant ethical regulations.

### DaPars2 analyses

Our previously developed DaPars2 software^40^, which allows the joint analyses of multiple samples based on a two-normal mixture model, was used to calculate the PDUI value. Briefly, we extract a 3′UTR annotation for each gene using bedtools^93^ and the “DaPars_Extract_Anno.py” script within DaPars2^40^. We then used samtools^94^ to calculate the sequencing depth for each sample. Finally, we used DaPars2^40^ to calculate the 3′UTR usage (PDUI value) of each transcript across samples.

### 3′aQTL mapping

As we previously described^37^, we applied a linear regression framework in Matrix eQTL^42^ to test the association between the normalized PDUI values and SNPs within an interval of 1 Mbp from the 3′UTR region, adjusting for known covariates (including sex, RIN, platform, post-mortem interval, and top five genotype PCs) and hidden covariates calculated by PEER^41^. The number of PEER covariates for each tissue was determined as suggested by the GTEx Consortium. We performed 1000 rounds of permutation to obtain empirical P values for each gene, which were then adjusted using the R package qvalue^95^.

### Building 3′aTWAS prediction models

We used transcriptome and individual-matched WGS genotype data from the ROS/MAP, PsychENCODE, and GTEx Consortia to establish 3′aTWAS single-tissue prediction models for 3′UTR usage (PDUI value) by FUSION^32^. Known covariates, including sex and genotyping platforms, post-mortem interval, and hidden batch effects or other unobserved covariates from PEER^41^ were used to residualized PDUI values calculated by Dapars2^40^. Residualized PDUI values were used to train cross-tissue 3′aTWAS models with genotype data. Prediction models with a heritability of Bonferroni-corrected *P* < 0.05 were used for 3′aTWAS analysis.

### Applying 3′aTWAS prediction models to GWAS summary statistics

We selected and downloaded GWAS summary statistics of 11 brain disorders (ALS^47^, ADHD^14^, ASD^15^, ANX^48^, BIP^96^, DEP^48^, MD^17^, SCZ^18^, PTSD^49^, PD^50^, and AD^97^), which were sufficiently powered to observe genome-wide significant SNPs. For PD and BIP, we used the publicly available summary statistics, excluding the 23andme samples. To facilitate and enhance comparisons with expression and splicing TWAS, we also selected 49 sufficiently powered summary statistics of psychiatric traits or non-brain-associated diseases. Variants in GWAS summary statistics were filtered to remove variants with minor allele frequency ≤ 0.01, indels, and all variants with ambiguous ref/alt alleles using ldsc software^98^. We then applied 3′aTWAS prediction models to filtered GWAS summary statistics data.

### Testing the enrichment of GWAS signals in 3′aQTL

We extracted 3′aQTLs (*P* < 1 × 10^−5^) that also belonged to psychiatric disorder GWAS SNPs and plotted the quantile–quantile plot (QQ plot) of GWAS *P* values for those SNPs. Similarly, we also generated the QQ plots for eQTLs and sQTLs (*P* < 1 × 10^−5^), and raw GWAS P values were plotted as a control for comparison. Following previously described strategies^99,100^, we applied GARFIELD^46^ to test the enrichment of brain disorder GWAS SNPs among 3′aQTLs. GARFIELD is a tool for assessing the enrichment of a complex trait and the overlapping functional features while controlling for LD, allele frequency and distance to genes^46^.

### Joint and conditional analysis

Joint and conditional testing was performed for 3′aTWAS-significant associations (FDR < 0.05) signals using FUSION^32^ to determine how much GWAS signal remained after the association from 3′aTWAS was removed. Briefly, a permutation test (*n* = 100,000) was conducted for each 3′aTWAS-significant association to shuffle the 3′aQTL weights and empirically determine an association statistic. 3′aTWAS-significant loci that pass the joint and conditional testing indicate that 3′UTR usage heterogeneity was captured and is less likely to be colocalized by chance.

### Expression TWAS and splicing TWAS analyses

For comparison, we performed expression TWAS and splicing TWAS analyses on the same GWAS summary statistics of brain disorders. The expression and splicing TWAS modules of GTEx v8 were obtained from PredictDB^101^.

### Colocalization of 3′aQTL and GWAS associations

Colocalization of 3′aQTL and GWAS associations was conducted using the coloc software^53^, which has been incorporated into the TWAS/FUSION pipeline^32^, using the default parameters for 3′aTWAS-significant associations. Briefly, coloc uses the approximate Bayes factor test approach and calculates five posterior probabilities (PP0–PP4) for the corresponding hypotheses: PP0, the posterior probabilities of the null model of no association; PP1, the posterior probabilities that causal SNPs are associated with GWAS signals only; PP2, the posterior probabilities that causal SNPs are associated with 3′aQTLs only; PP3, the posterior probabilities that causal SNPs of GWAS signals and 3′aQTLs are independent; PP4, the posterior probabilities of GWAS signals and 3′aQTLs share causal SNPs. We consider PP4 ≥ 0.5 to represent significant colocalization.

### 3′aQTL and 3′aTWAS fine-mapping analyses

We used the fine-mapping tool SuSiE^54^ to identify potentially causal SNPs of 3′aQTLs. Only the 3′aQTLs of significant 3′aTWAS genes were included in the fine-mapping analyses. 3′aTWAS fine-mapping analyses was performed by FOCUS^52^ using the same reference panels of 3′aTWAS analyses.

### Cell culture

HEK293T cells (human embryonic kidney cells, from ATCC) were maintained in Dulbecco’s modified Eagle medium (ThermoFisher, 11965084) supplemented with 10% fetal bovine serum and 50 U/mL penicillin-streptomycin. SH-SY5Y cells (human neuroblastoma cells, from ATCC) were maintained in 50% EMEM (ATCC, 30-2003), 50% Ham’s F12 (ThermoFisher, 11765047), supplemented with 10% fetal bovine serum, and 50 U/mL penicillin–streptomycin. Cells were grown at 37 °C and 5% CO_2_.

### qRT-PCR

HEK293T cells were transfected with scramble shRNA or *ATXN3* shRNAs (Sigma) for 48 h prior to RNA isolation (Qiagen, 74106) and cDNA synthesis (ThermoFisher, 11756500). Relative fold change of *ATXN3* was determined by qRT-PCR using SYBR Green Master Mix (ThermoFisher, A25776) with *GAPDH* as an endogenous control.

*ATXN3* shRNAs (Sigma, NM_004993) were obtained in the MISSION® pLKO.1 backbone plasmid. Target sequences:

#1 - TRCN0000007405 - CGTCGGTTGTAGGACTAAATA (3’UTR)

#2 - TRCN0000007406 - CGAGTGTTAGAAGCAAATGAT (CDS)

#3 - TRCN0000007407 - GCAGGGCTATTCAGCTAAGTA (CDS)

Primers:

*ATXN3* fwd: 5’-TCGGAAGAGACGAGAAGCCTAC-3’

*ATXN3* rev: 5’-AAGTGCTCCTGAACTGGTGGCT-3’

*GAPDH* fwd: 5’-CGCTCTCTGCTCCTCCTGTT-3’

*GAPDH* rev: 5’-CCATGGTGTCTGAGCGATGT-3’

### Western blot

Protein extracts were prepared in radioimmunoprecipitation assay lysis buffer (ThermoFisher, 89900) with protease and phosphatase inhibitors (ThermoFisher, 87786) (note: lysates were not centrifuged). Lysates were sonicated at 4 °C (Bioruptor Pico), denatured in LDS sample buffer (ThermoFisher, NP0007) with a reducing agent (ThermoFisher, NP0009), and heated at 70 °C for 10 min. Lysates were electrophoresed by sodium dodecyl sulfate–polyacrylamide gel electrophoresis using a 4%–20% gradient polyacrylamide gel (Bio-Rad, 5678093) and transferred to 0.45-μm polyvinylidene difluoride membrane (Bio-Rad, 1704157). Blots were incubated with primary antibodies diluted in 5% nonfat milk in TBST (TRIS-buffered saline, 0.05% Tween-20) overnight at 4 °C and with secondary antibodies diluted in 5% nonfat milk in TBST (ThermoFisher, A16078 [goat anti-mouse HRP, 1:3000], A16110 [goat anti-rabbit HRP, 1:3000], A32728 [goat anti-mouse Alexa Fluor^TM^ 647, 1:1000], A32733 [goat anti-rabbit Alexa Fluor^TM^ 647, 1:1000]) for 60 min at room temperature. Detection was performed using enhanced chemiluminescence substrate (Genesee Scientific, 20–300S) and imaged on a ChemiDoc MP (Bio-Rad). Primary antibodies used in this study were TDP-43 (Proteintech, 12892-1-AP, 1:3000), phospho-TDP-43 S409/S410 (Proteintech, 22309-1-AP, 1:3000), ATXN3 (Millipore-Sigma, MAB5360, clone 1H9, 1:2000), and α-tubulin (ThermoFisher, 62204, 1:3000).

### Cycloheximide chase experiment

HEK293T cells were transfected with scramble shRNA or *ATXN3* shRNA #1 along with mCherry-TDP-43 CTF for 24 h. Fresh media was added containing 20 µg/mL cycloheximide (Sigma, C4859) to inhibit protein synthesis. Cells were harvested at 0, 4, 8, or 12 h and analyzed by western blot.

### PPI network and pathway analysis

A unified web platform inBio Discover^62^ was used to evaluate the enrichment of non-HLA 3′aTWAS genes in known disease gene lists (defined by inBio Discover) and pathways. We also used STRING^102^ and inBio Discover^62^ to evaluate the interconnectivity of non-HLA 3′aTWAS genes by physical PPIs. inBio Discover^62^ collected PPIs from the InWeb3 database, which contains 420,000 interactions between 12,793 human proteins. Cytoscape (v3.8.0)^103^ was used to visualize the PPI network and the enriched diseases and pathways of 3′aTWAS genes.

### Reporting summary

Further information on research design is available in the Nature Portfolio Reporting Summary linked to this article.

## Supplementary information

Supplementary Information

Description of Additional Supplementary Files

Supplementary Data 1

Supplementary Data 2

Supplementary Data 3

Reporting Summary

## Data Availability

No new sequencing data were created in this study. The raw RNA-seq and genotype data of the GTEx cohort are available to authorized users through dbGaP release, under accession code phs000424.v8.p2. The data are available under controlled access due to data privacy laws. Data access application review time depends on the dbGaP database. The raw RNA-seq and genotype of the ROS/MAP cohort are available in the AD Knowledge Portal under accession code syn3219045. The data are available under controlled access due to data privacy laws. To access the ROS/MAP cohort, users need to complete and submit a signed Data Use Certificate (DUC) to the AD Knowledge Portal at https://adknowledgeportal.synapse.org/Data%20Access. The DUC must include the objectives of the proposed research, study design and analysis plan. The AD Knowledge Portal will review the application and the expected review time is about two weeks. The raw RNA-seq and genotype data of the PsychENCODE cohort are available in the PsychENCODE Knowledge Portal under accession code syn4921369. The data are available under controlled access due to data privacy laws. To access the PychENCODE cohort, users need to submit an online request access form with a signed Distribution Agreement [https://www.nimhgenetics.org/data/request-access/distribution-agreement.pdf] to NIMH Repository & Genomics Resource at https://www.nimhgenetics.org/request-access/how-to-request-access. All requests for access to PychENCODE cohort data will be reviewed by a trans-NIH Data Access Committee consisting of NIH Program Staff from NIDA, NIAAA, and NIMH. Expected data access application review time is about three months. The processed 3′aQTL summary statistics, 3′aTWAS models and all significant 3′aTWAS genes in 11 brain disorders are freely available at https://wlcb.oit.uci.edu/3aTWAS and Synapse (accession no. syn50919268). All significant 3′aTWAS genes in 11 brain disorders are also available at Supplementary Data 1. The expression and splicing TWAS modules of GTEx v8 in PredictDB are publicly available at https://predictdb.org/. The details, including accession numbers, of GWAS summary statistics used in this study are listed in Supplementary Table 1. PTSD GWAS summary statistic is available to authorized users through dbGaP release, under accession code phs001672.v9.p1. Other GWAS summary statistics are publicly available at GWAS catalog [https://www.ebi.ac.uk/gwas/]. Source data for uncropped blots are provided with this paper. Source data are provided with this paper.

## Source data

Source Data
