# Supplementary material for: Alternative polyadenylation transcriptome-wide association study identifies APA-linked susceptibility genes in brain disorders
Source: Nat Commun. 2023 Feb 3;14:583. doi: 10.1038/s41467-023-36311-8 (PMC9898543; doi:10.1038/s41467-023-36311-8)
Supplement: Supplementary file 1 — Supplementary Information [file 41467_2023_36311_MOESM1_ESM.pdf]

Supplementary Information

Alternative polyadenylation transcriptome-wide association study identifies APA-linked susceptibility genes in brain disorders

Ya Cui[1], Frederick J. Arnold[2], Fanglue Peng[3], Dan Wang[4], Jason Sheng Li[1], Sebastian Michels[2], Eric J. Wagner[5], Albert R. La Spada[2]*, Wei Li[1]*

[1]Division of Computational Biomedicine, Department of Biological Chemistry, School of Medicine, University of California, Irvine, Irvine, CA 92697, USA

[2]Departments of Pathology & Laboratory Medicine, Neurology, and Biological Chemistry, School of Medicine, and the UCI Institute for Neurotherapeutics, University of California Irvine, Irvine, CA 92697, USA

[3]Department of Molecular and Cellular Biology, Baylor College of Medicine, Houston, TX 77030, USA

[4]Department of Medicine, Division of Cardiology, University of California, Los Angeles, Los Angeles, CA, 90095, USA

[5]School of Medicine and Dentistry, University of Rochester Medical Center, Rochester, NY 14642, USA

These authors contributed equally: Ya Cui, Frederick J. Arnold.

*Corresponding authors: alaspada@hs.uci.edu (A.R.L.S.) and wei.li@uci.edu (W. L.)

Supplementary Figures

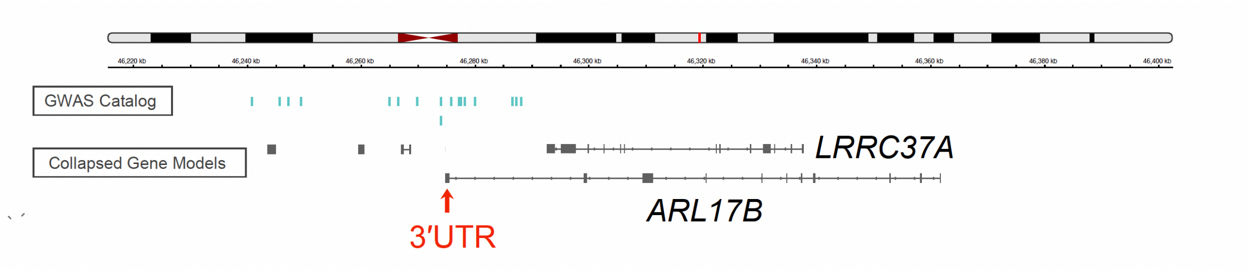

Supplementary Figure 1. Integrative Genomics Viewer (IGV) plot in the *ARL17B* locus. GWAS lead SNPs are located near the *ARL17B* 3′ UTR. The first track shows GWAS lead SNPs from the GWAS Catalog[1]. The second track shows gene structures. The red arrow indicates the 3′UTR region of *ARL17B*.

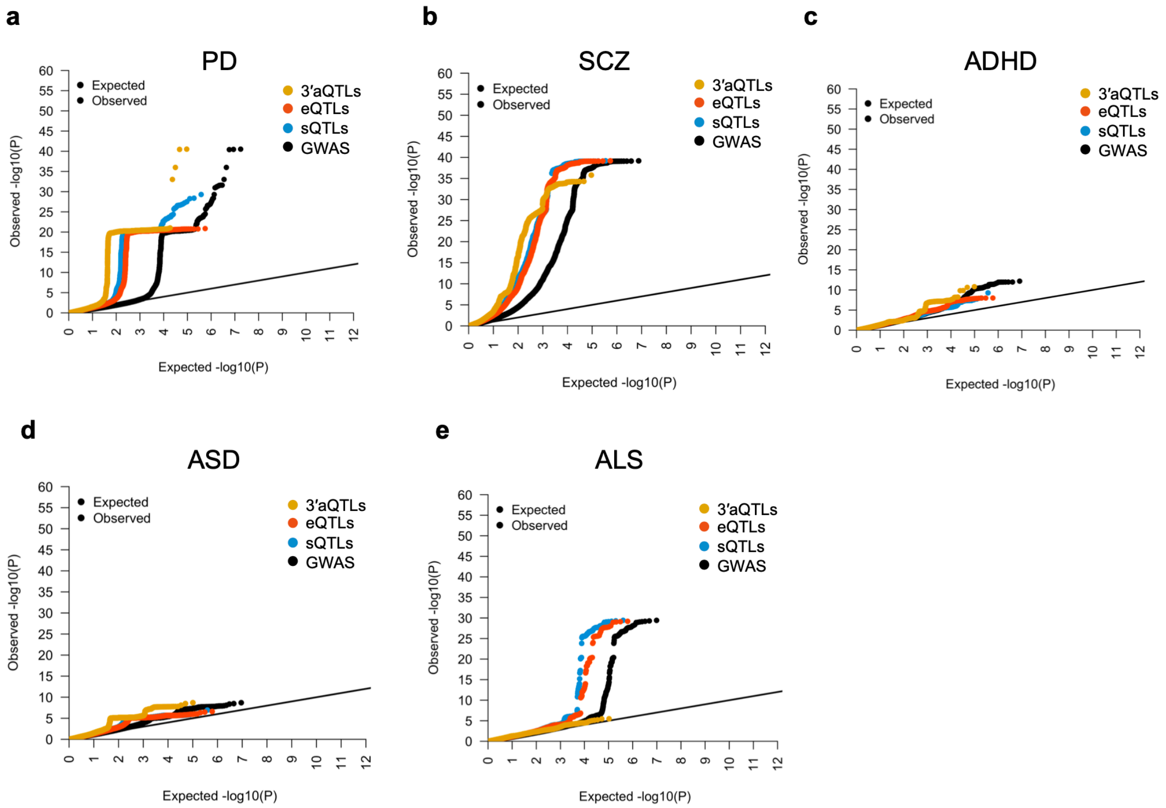

Supplementary Figure 2. Quantile-quantile plots of five brain disorders. a Quantile-quantile plots showing the nominal P-value of PD' GWAS SNPs (black) which were binarily annotated by 3′aQTLs (yellow), sQTLs (blue) and eQTLs (light orange) with nominal P-value < 10^{-5}. **b** Similar to (a) for the SCZ' GWAS SNPs. **c** Similar to (a) for the ADHD' GWAS SNPs. **d** Similar to (a) for the ASD' GWAS SNPs. **e** Similar to (a) for the ALS' GWAS SNPs. PD represents Parkinson's disease. SCZ represents schizophrenia. ADHD represents attention deficit hyperactivity disorder. ASD represents autism spectrum disorder. ALS represents amyotrophic lateral sclerosis.

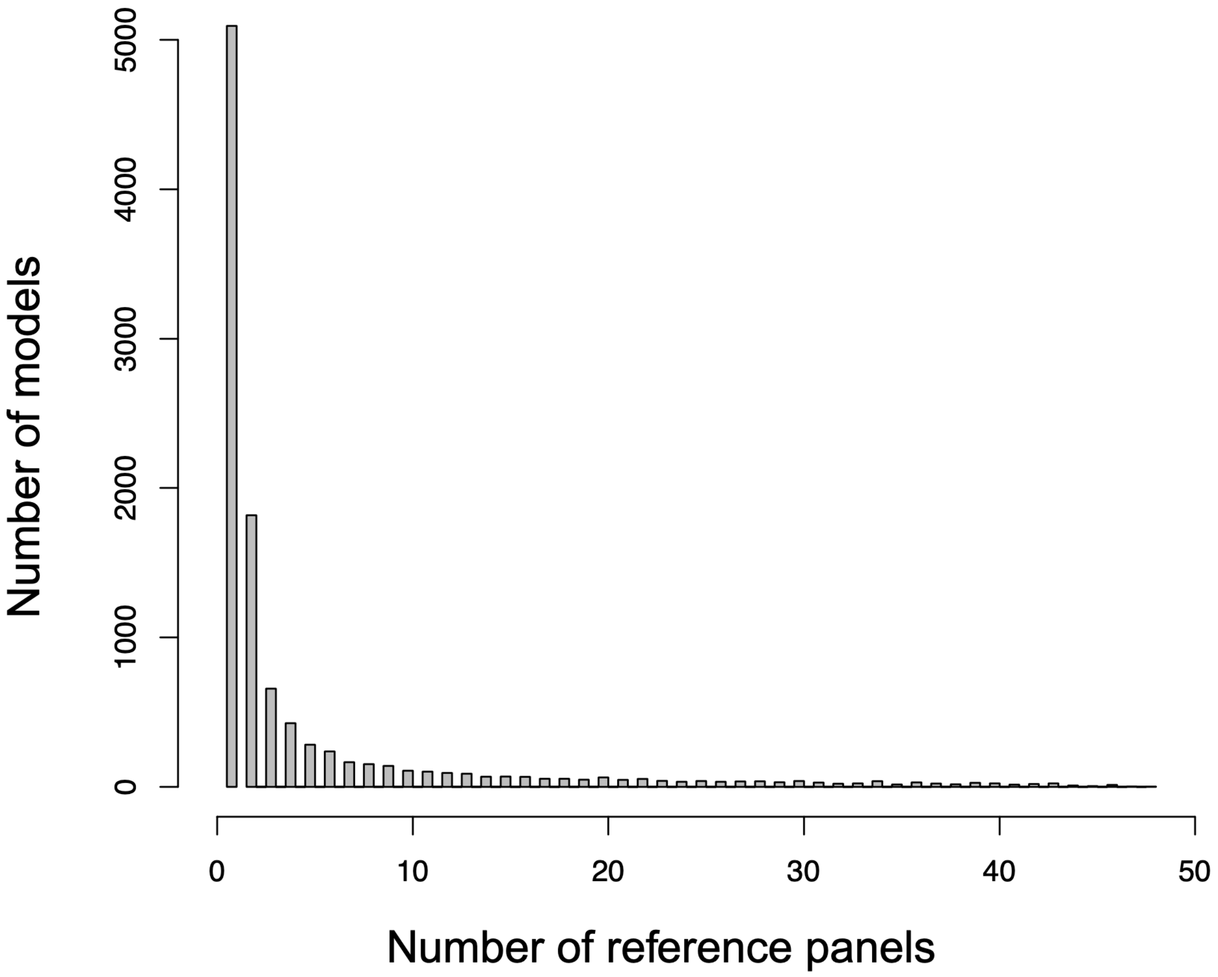

Supplementary Figure 3. Overlap of 3′aTWAS models in reference panels. The x-axis indicates the number of overlap reference panels of a 3′aTWAS model. The y-axis indicates the number of 3′aTWAS models.

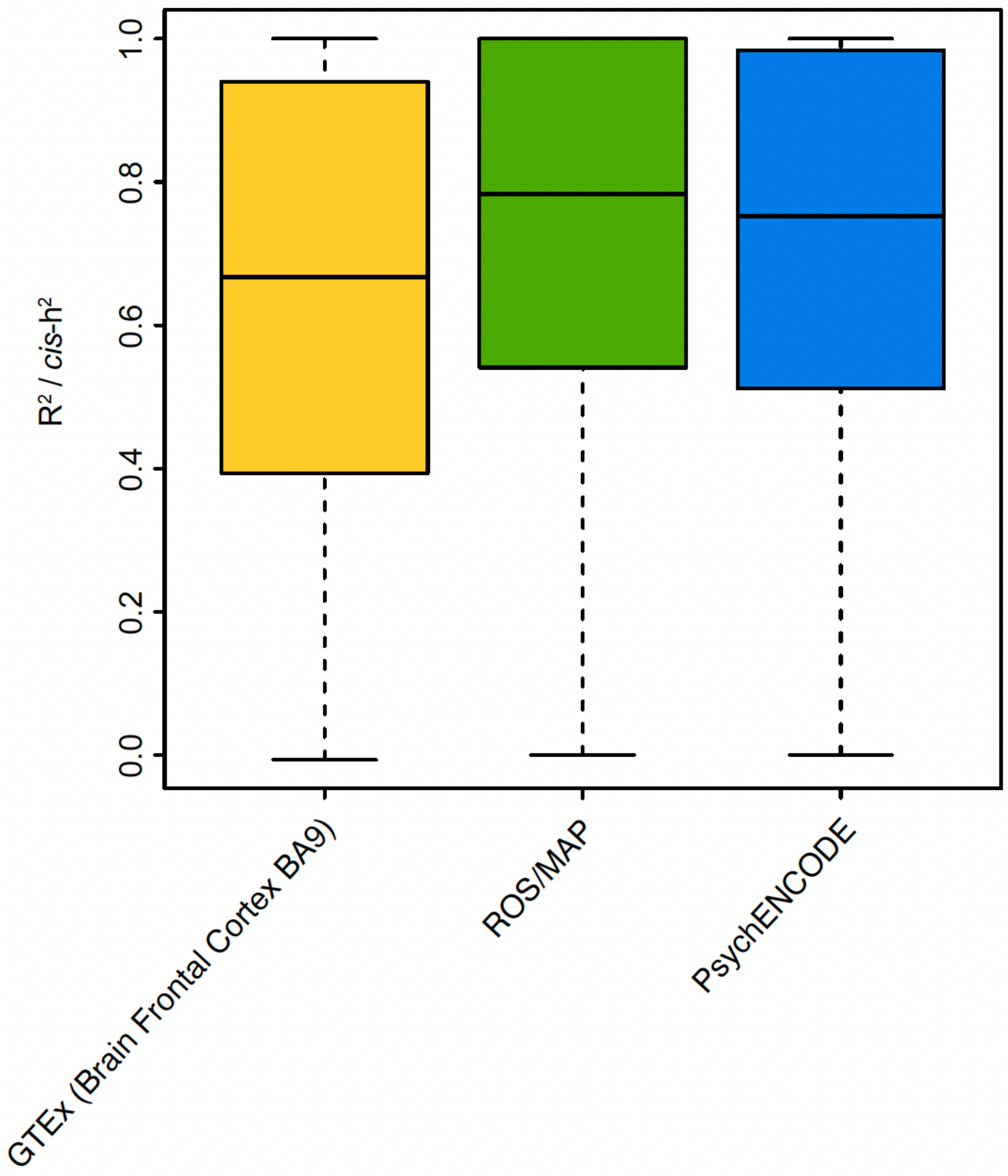

Supplementary Figure 4. Boxplot of prediction accuracy for 3′aTWAS models. The average in-sample prediction accuracy (measured by heritability normalized R^2, R^2/*cis*-h^2) of 3′ aTWAS models in ROS/MAP (n = 1,837), PsychENCODE (n = 2,064) and GTEx Frontal Cortex BA9 (n = 606) cohorts. The center horizontal lines within the plot represent the median values and the boxes are bounded by the 25th and 75th percentile. The whiskers extend to the maximum and minimum values within 1.5 times of the interquartile range.

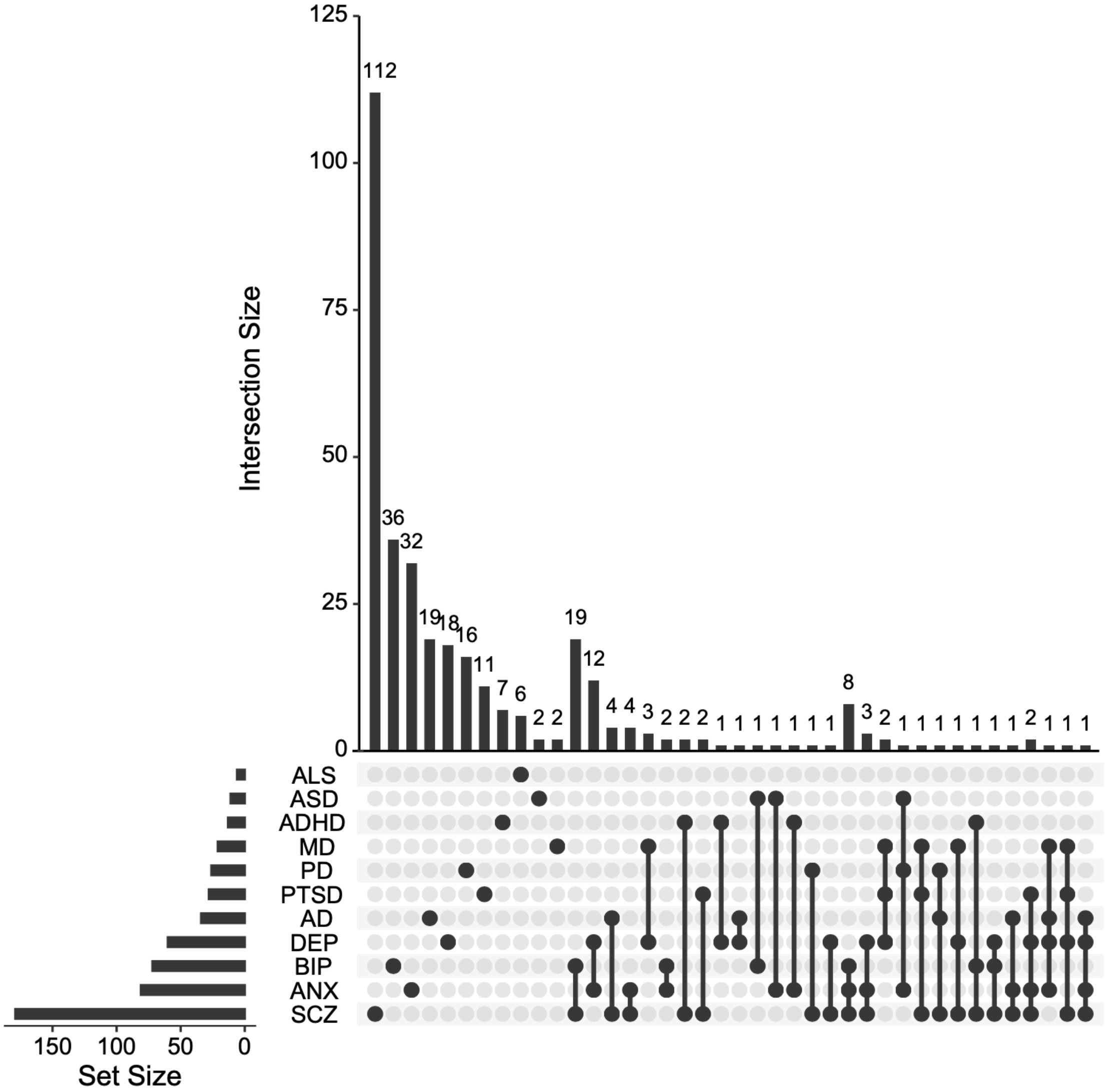

Supplementary Figure 5. Upset plot shows the number of cross-brain disorder 3′aTWAS significant genes. The number of 3′aTWAS significant genes for 11 brain disorders are indicated on the x-axis and sorted by increasing set size. The intersection size corresponds with the number of shared 3′aTWAS significant genes identified in different brain disorders. ALS represents amyotrophic lateral sclerosis. ADHD represents attention deficit hyperactivity disorder. ASD represents autism spectrum disorder. ANX represents anxiety. BIP represents bipolar disorder. DEP represents depression. MDD represents major depressive disorder. SCZ represents schizophrenia. PTSD represents post-traumatic stress disorder. PD represents Parkinson’s disease. AD represents Alzheimer’s disease.

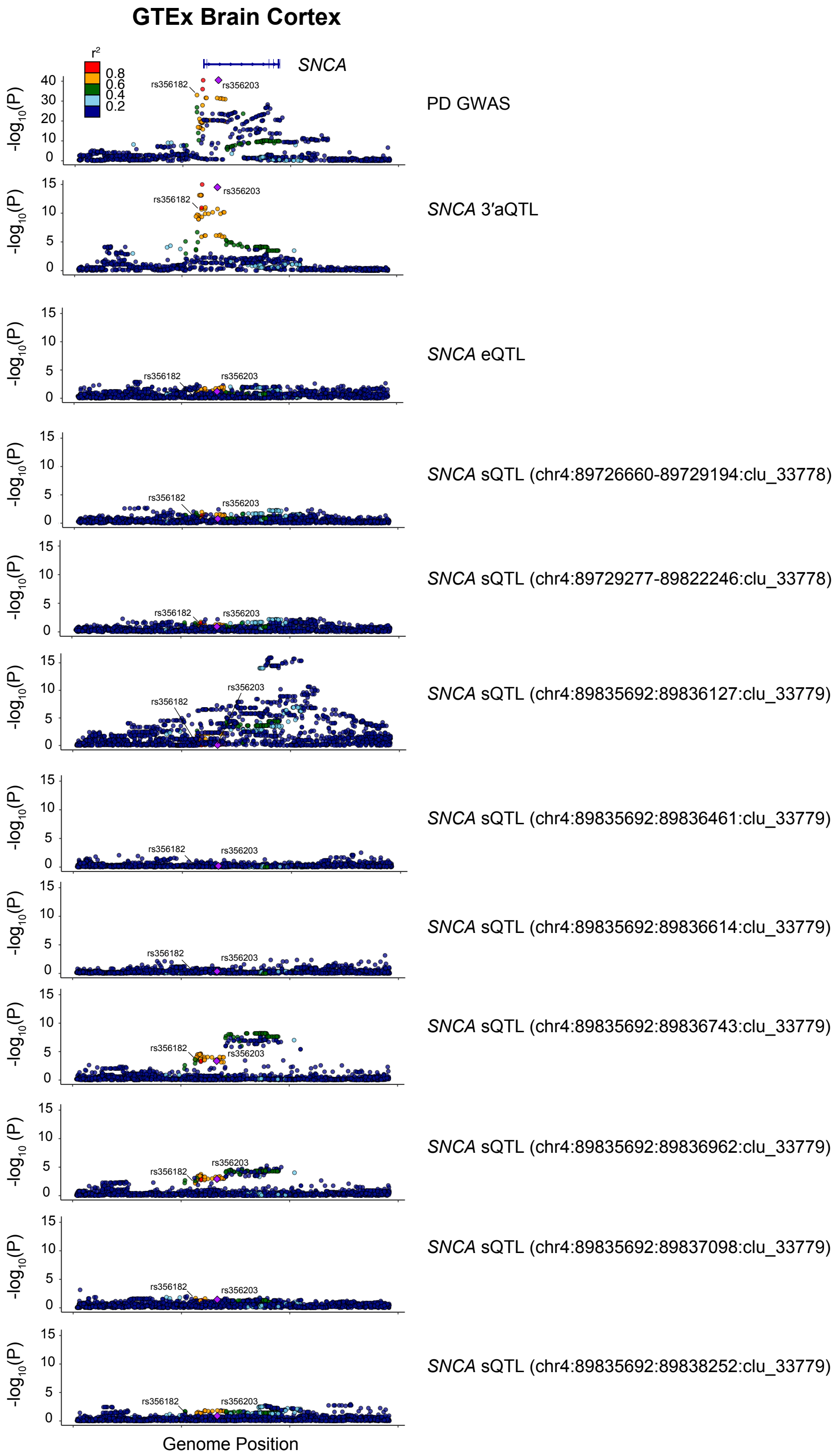

Supplementary Figure 6. Aligned Manhattan plots of Parkinson's disease GWAS and QTLs (3′aQTLs, eQTLs and sQTLs) from GTEx brain cortex tissue at the *SNCA* locus. Please note that rs356203 is the lead PD GWAS SNP without including 23andMe samples and rs356182 is the lead PD GWAS SNP including 23andMe samples. SNPs are colored by LD (r^2). PD represents Parkinson's disease.

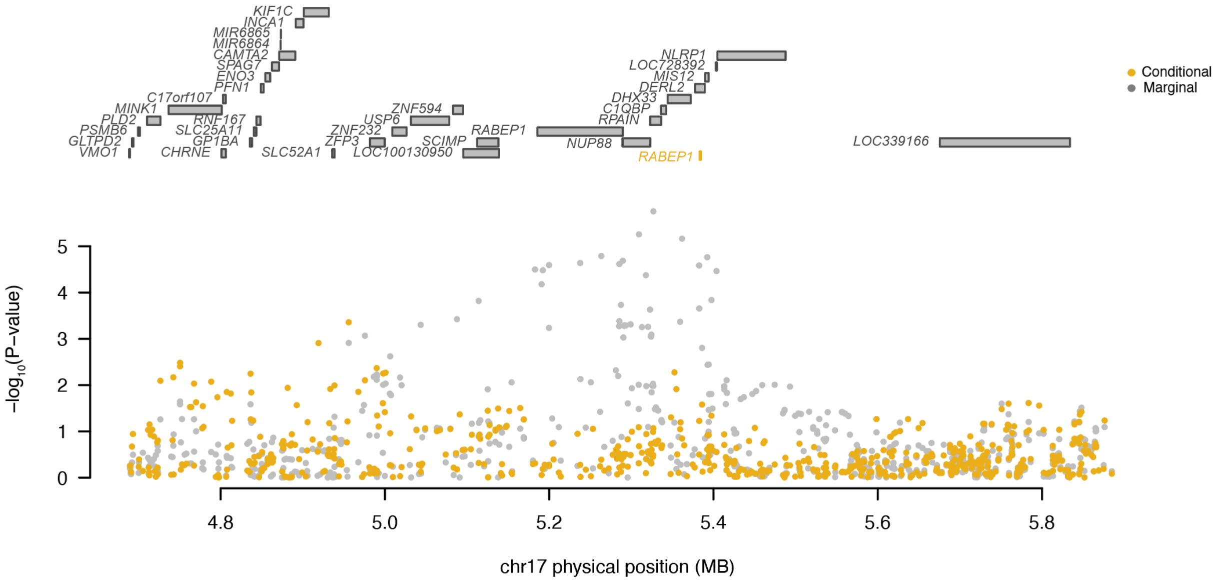

Supplementary Figure 7. Regional association plot of *RABEP1* in SCZ. SCZ GWAS signal at the *RABEP1* locus (gray) and GWAS signal after removing the effects of *RABEP1* 3′UTR usage (yellow). SCZ represents schizophrenia.

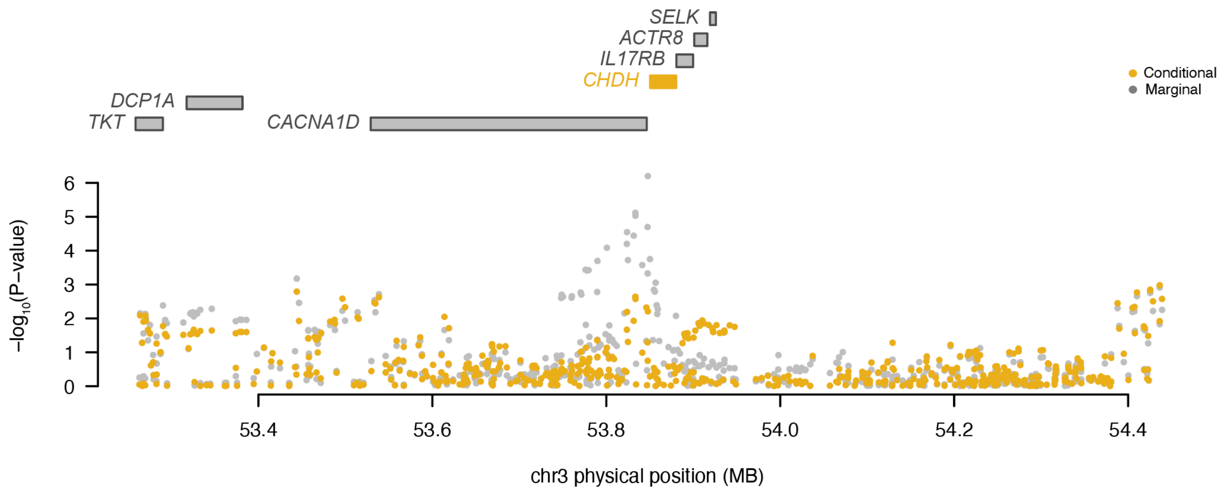

Supplementary Figure 8. Regional association plot of *CHDH* in BIP. BIP GWAS signal at the *CHDH* locus (gray) and GWAS signal after removing the effects of *CHDH* 3′UTR usage (yellow). BIP represents bipolar disorder.

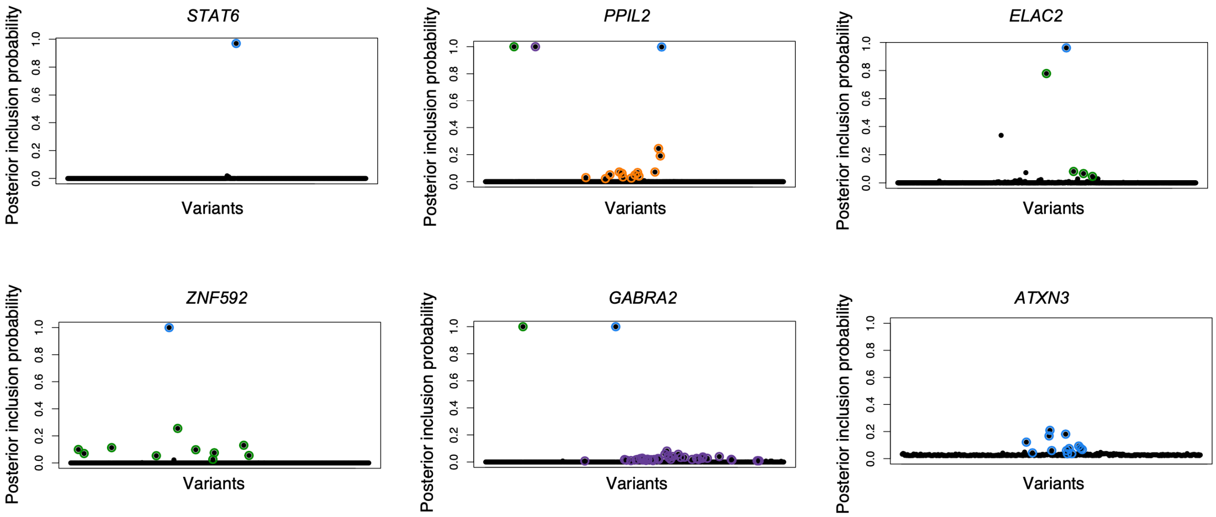

Supplementary Figure 9. 3'aQTL fine-mapping results of *STAT6*, *PPIL2*, *ELAC2*, *ZNF592*, *GABRA2* and *ATXN3*. The x-axis indicates variants at each locus. The y-axis indicates the fine-mapping posterior inclusion probability. Each point represents a SNP. SNPs with the same color circles indicates that these SNPs are in the same credible set.

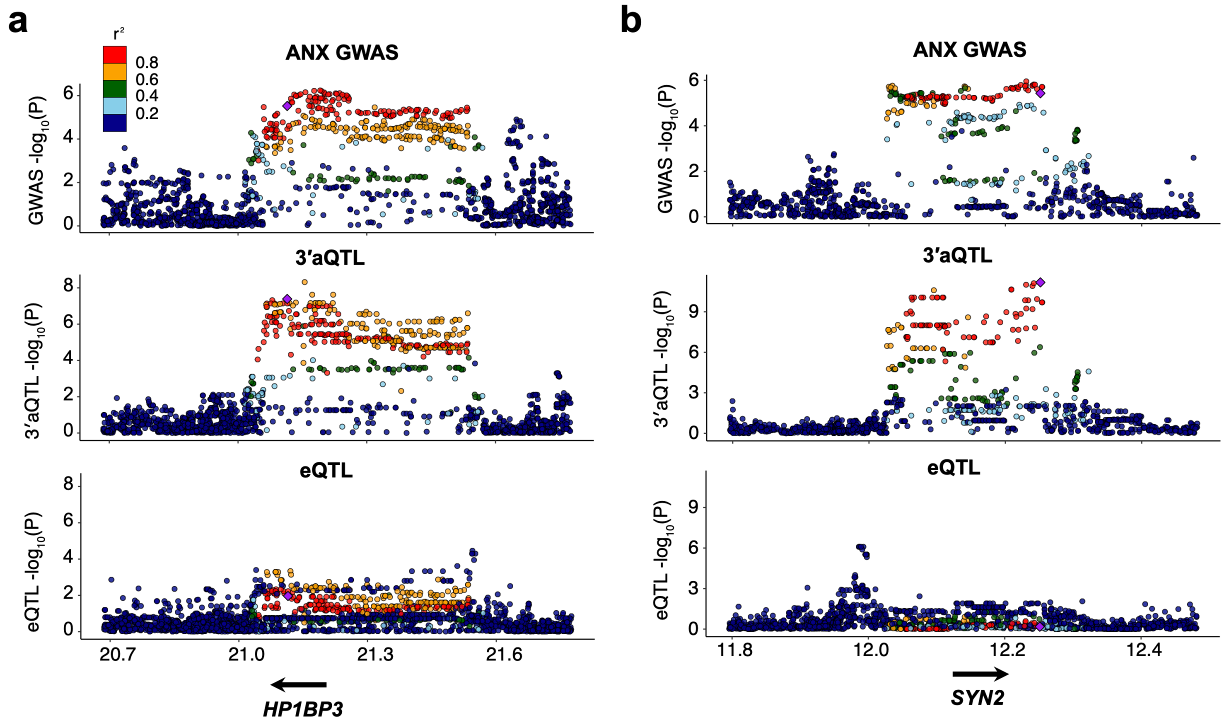

Supplementary Figure 10. Aligned Manhattan plots at *HP1BP3* and *SYN2* loci. a Aligned Manhattan plots of ANX (anxiety) GWAS and QTLs (3′aQTLs and eQTLs) from GTEx brain cerebellum tissue at the *HP1BP3* locus, and **b** GTEx brain cerebellar hemisphere tissue at the *SYN2* locus. SNPs are colored by LD (r^2).

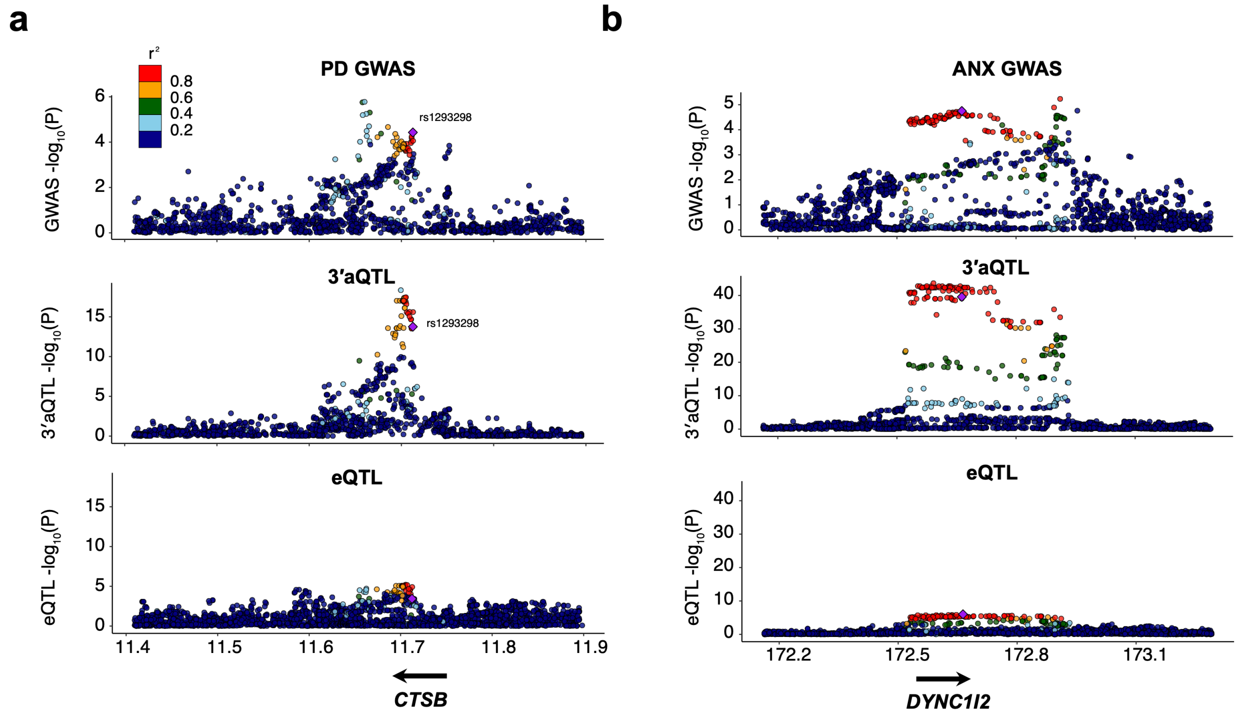

Supplementary Figure 11. Aligned Manhattan plots at *CTSB* and *DYNC1I2* loci. a Aligned Manhattan plots of PD (Parkinson's disease) GWAS and QTLs (3′aQTLs and eQTLs) from GTEx brain cortex tissue at the *CTSB* locus. **b** Aligned Manhattan plots of ANX (anxiety) GWAS and QTLs (3′aQTLs and eQTLs) from GTEx brain cerebellum tissue at the *DYNC1I2* locus. SNPs are colored by LD (r^2).

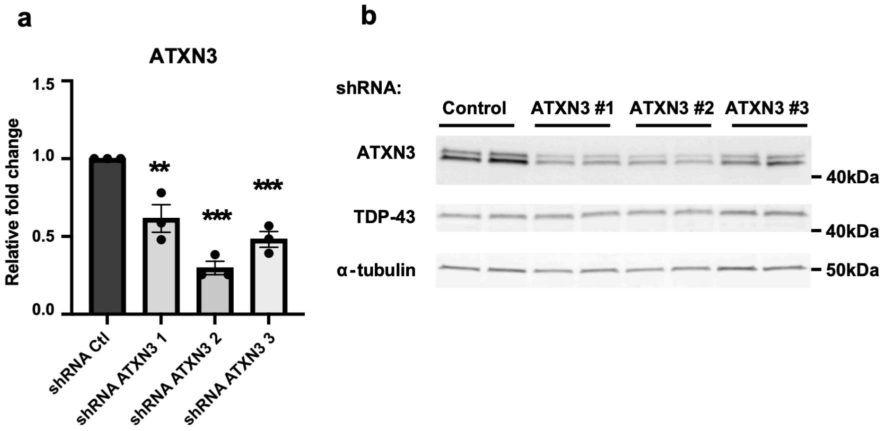

Supplementary Figure 12. Validation of shRNAs targeting human *ATXN3*. a Validation of *ATXN3* knockdown by three independent shRNAs targeting human *ATXN3* relative to shRNA control by qRT-PCR ($p = 0.0030$, $p = 0.0001$, $p = 0.0004$ for *ATXN3* shRNA #1, #2, or #3, respectively). This experiment was repeated $n = 3$ times. **b** Western analysis of ATXN3 in HEK293T cells transfected with shRNAs for 48 hrs. **c** Quantification of ATXN3 knockdown in cells transfected with shRNA ATXN3 #1 ($p = 0.0371$), #2 ($p = 0.0129$), or #3 ($p = 0.0504$) relative to shRNA control. This experiment was repeated $n = 4$ times. Data are presented as mean values +/- SEM. Statistical significance was determined by one-way ANOVA with Dunnett's multiple comparisons test. * represents $p < 0.05$. ** represents $p < 0.01$. *** represents $p < 0.001$.

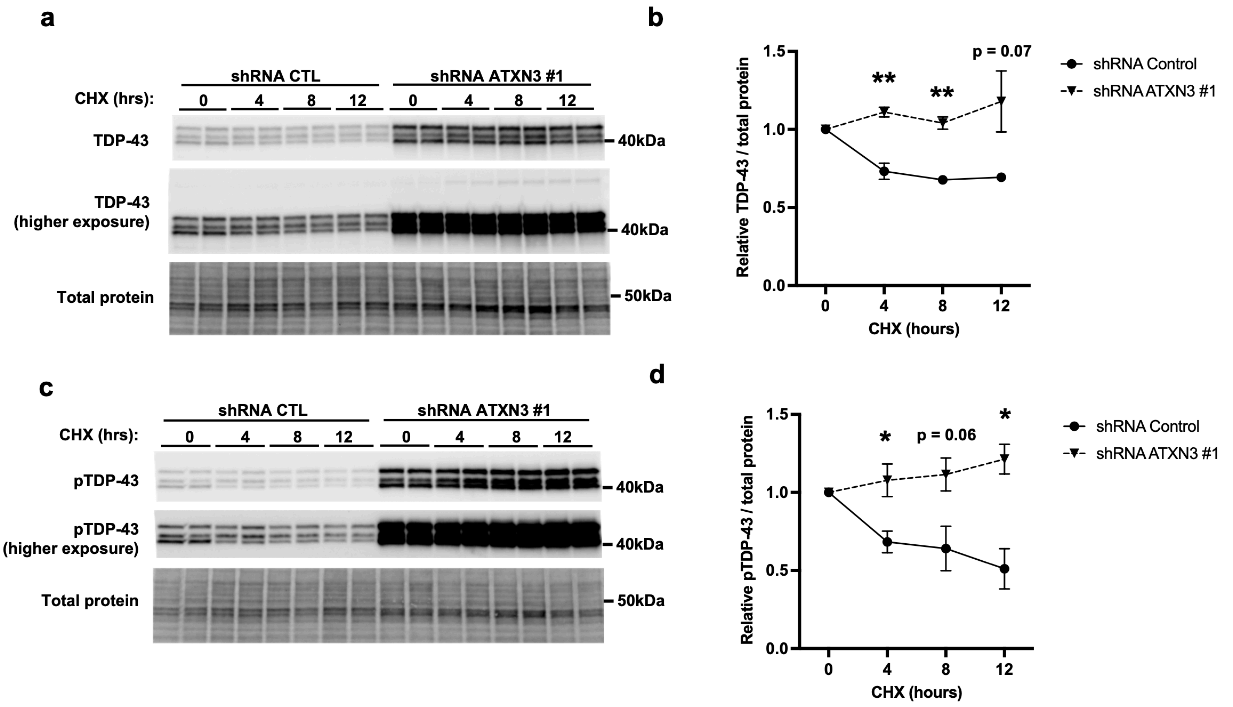

Supplementary Figure 13. Knockdown of *ATXN3* increases the stability of TDP-43 CTF. a Western analysis of total TDP-43. HEK293T cells were transfected with mCherry-tagged TDP-43 CTF and shRNAs for 24 hrs then treated with 20 µg/mL cycloheximide (CHX) for the indicated time-course. **b** Quantification of total TDP-43 in cells treated with CHX for 4 hrs ($p = 0.0034$), 8 hrs ($p = 0.0013$), or 12 hrs ($p = 0.0689$) normalized to 0 hrs. **c** Western analysis of pTDP-43 (S409/S410). HEK293T cells were transfected with mCherry-tagged TDP-43 CTF and shRNAs for 24 hrs then treated with 20 µg/mL cycloheximide (CHX) for the indicated time-course. **d** Quantification of pTDP-43 in cells treated CHX for 4 hrs ($p = 0.0350$), 8 hrs ($p = 0.0561$), or 12 hrs ($p = 0.0119$) normalized to 0 hrs. Each experiment was repeated $n = 3$ times. Data are presented as mean values +/- SEM. Statistical significance was determined by unpaired two-tailed t-test at each time point. * represents $p < 0.05$. ** represents $p < 0.01$.

Supplementary Tables

Supplementary Table 1. Details on GWAS data used in this study						
Num	Disease	Year	Journal	Pubmed ID	Source	Accession number
1	Parkinson's disease (PD)	2019	Lancet Neurol	31701892[2]	GWAS catalog	GCST009325
2	Schizophrenia (SCZ)	2022	Nature	35396580[3]	GWAS catalog	GCST90128471
3	Attention-deficit hyperactivity disorder (ADHD)	2018	Nat Genet	30478444[4]	GWAS catalog	GCST007543
4	Autism spectrum disorder (ASD)	2019	Nat Genet	30804558[5]	GWAS catalog	GCST007556
5	Amyotrophic lateral sclerosis (ALS)	2016	Nat Genet	27455348[6]	GWAS catalog	GCST004692
6	Anxiety (ANX)	2021	Nat Hum Behav	33859377[7]	GWAS catalog	GCST012354
7	Depression (DEP)	2021	Nat Hum Behav	33859377[7]	GWAS catalog	GCST012355
8	Major depressive disorder (MDD)	2021	Nat Neurosci	34045744[8]	GWAS catalog	GCST90020222
9	Post-traumatic stress disorder (PTSD)	2021	Nat Genet	33510476[9]	dbGaP	phs001672.v9.p1
10	Alzheimer's disease (AD)	2021	Nat Genet	34493870[10]	GWAS catalog	GCST90044699
11	Bipolar disorder (BIP)	2021	Nat Genet	34002096[11]	GWAS catalog	GCST012465

Supplementary Table 2. 3'aTWAS genes of ALS				
Num	Disease	Transcript ID	Gene Symbol	Colocalization Probability (PP4)
1	ALS	NR_028466	ATXN3	0.969
2	ALS	NM_145005	C9orf72	0.968
3	ALS	NR_033936	GOLGA2P10	0.916

4	ALS	NR_102747	LOC727751	0.916
5	ALS	NR_102748	LOC727751	0.916
6	ALS	NR_026811	GOLGA2P10	0.914
7	ALS	NM_145005	C9orf72	0.302
8	ALS	NM_001365819	SBF1	0.29
9	ALS	NM_001190991	CNPY2	0.056

Supplementary References

1. Buniello A*, et al.* The NHGRI-EBI GWAS Catalog of published genome-wide association studies, targeted arrays and summary statistics 2019. *Nucleic Acids Res* **47**, D1005-D1012 (2019).

2. Nalls MA*, et al.* Identification of novel risk loci, causal insights, and heritable risk for Parkinson's disease: a meta-analysis of genome-wide association studies. *Lancet Neurol* **18**, 1091-1102 (2019).

3. Trubetskoy V*, et al.* Mapping genomic loci implicates genes and synaptic biology in schizophrenia. *Nature* **604**, 502-508 (2022).

4. Demontis D*, et al.* Discovery of the first genome-wide significant risk loci for attention deficit/hyperactivity disorder. *Nat Genet* **51**, 63-75 (2019).

5. Grove J*, et al.* Identification of common genetic risk variants for autism spectrum disorder. *Nat Genet* **51**, 431-444 (2019).

6. van Rheenen W*, et al.* Genome-wide association analyses identify new risk variants and the genetic architecture of amyotrophic lateral sclerosis. *Nat Genet* **48**, 1043-1048 (2016).

7. Thorp JG*, et al.* Symptom-level modelling unravels the shared genetic architecture of anxiety and depression. *Nat Hum Behav* **5**, 1432-1442 (2021).

8. Levey DF*, et al.* Bi-ancestral depression GWAS in the Million Veteran Program and meta-analysis in >1.2 million individuals highlight new therapeutic directions. *Nat Neurosci* **24**, 954-963 (2021).

9. Stein MB*, et al.* Genome-wide association analyses of post-traumatic stress disorder and its symptom subdomains in the Million Veteran Program. *Nat Genet* **53**, 174-184 (2021).

10. Wightman DP*, et al.* A genome-wide association study with 1,126,563 individuals identifies new risk loci for Alzheimer's disease. *Nat Genet* **53**, 1276-1282 (2021).

11. Mullins N*, et al.* Genome-wide association study of more than 40,000 bipolar disorder cases provides new insights into the underlying biology. *Nat Genet* **53**, 817-829 (2021).
