# Supplementary material for: Alternative polyadenylation transcriptome-wide association study identifies APA-linked susceptibility genes in brain disorders
Source: Nat Commun. 2023 Feb 3;14:583. doi: 10.1038/s41467-023-36311-8 (PMC9898543; doi:10.1038/s41467-023-36311-8)
Supplement: Supplementary file 2 — Description of Additional Supplementary Files [file 41467_2023_36311_MOESM2_ESM.pdf]

**Description of Additional Supplementary Files**

**File Name:** Supplementary Data 1

**Description:** 3′aTWAS genes of 11 brain disorders.

**File Name:** Supplementary Data 2

**Description:** 3′aTWAS fine-mapping results of 11 brain disorders.

**File Name:** Supplementary Data 3

**Description:** 3′aQTLs fine-mapping results for significant 3′aTWAS genes of 11 brain disorders.
