# Supplementary material for: Alternative polyadenylation transcriptome-wide association study identifies APA-linked susceptibility genes in brain disorders
Source: Nat Commun. 2023 Feb 3;14:583. doi: 10.1038/s41467-023-36311-8 (PMC9898543; doi:10.1038/s41467-023-36311-8)
Supplement: Supplementary file 6 — Reporting Summary [file 41467_2023_36311_MOESM6_ESM.pdf]

nature portfolio

Corresponding author(s): Wei Li, Albert La Spada

Last updated by author(s): Dec 9, 2022

Reporting Summary

Nature Portfolio wishes to improve the reproducibility of the work that we publish. This form provides structure for consistency and transparency in reporting. For further information on Nature Portfolio policies, see our Editorial Policies and the Editorial Policy Checklist.

Statistics

For all statistical analyses, confirm that the following items are present in the figure legend, table legend, main text, or Methods section.

n/a	Confirmed	
☐	☒	The exact sample size (*n*) for each experimental group/condition, given as a discrete number and unit of measurement
☐	☒	A statement on whether measurements were taken from distinct samples or whether the same sample was measured repeatedly
☐	☒	The statistical test(s) used AND whether they are one- or two-sided<br>*Only common tests should be described solely by name; describe more complex techniques in the Methods section.*
☐	☒	A description of all covariates tested
☐	☒	A description of any assumptions or corrections, such as tests of normality and adjustment for multiple comparisons
☐	☒	A full description of the statistical parameters including central tendency (e.g. means) or other basic estimates (e.g. regression coefficient) AND variation (e.g. standard deviation) or associated estimates of uncertainty (e.g. confidence intervals)
☐	☒	For null hypothesis testing, the test statistic (e.g. *F*, *t*, *r*) with confidence intervals, effect sizes, degrees of freedom and *P* value noted<br>*Give P values as exact values whenever suitable.*
☒	☐	For Bayesian analysis, information on the choice of priors and Markov chain Monte Carlo settings
☒	☐	For hierarchical and complex designs, identification of the appropriate level for tests and full reporting of outcomes
☐	☒	Estimates of effect sizes (e.g. Cohen's *d*, Pearson's *r*), indicating how they were calculated

Our web collection on statistics for biologists contains articles on many of the points above.

Software and code

Policy information about availability of computer code

Data collection	No software was used.
Data analysis	R v3.6.1, STAR v2.5.3, Cytoscape v3.8.0, GARFIELD v2, Matrix eQTL v2.3, bedtools v2.17.0, PLINK v1.9, coloc 3.2-1, qvalue v2.22.0, ldsc v1.0.1, SuSiE v0.12.10, samtools v1.7, PEER v1.3, FOCUS v0.802, FUSION 2016 (https://github.com/gusevlab/fusion_twas), inBio Discover v2021-04-07 (https://inbio-discover.com), The open-source DaPars v2 program is freely available at https://github.com/3UTR/DaPars2.

For manuscripts utilizing custom algorithms or software that are central to the research but not yet described in published literature, software must be made available to editors and reviewers. We strongly encourage code deposition in a community repository (e.g. GitHub). See the Nature Portfolio guidelines for submitting code & software for further information.

Data

Policy information about availability of data

All manuscripts must include a data availability statement. This statement should provide the following information, where applicable:

- Accession codes, unique identifiers, or web links for publicly available datasets
- A description of any restrictions on data availability
- For clinical datasets or third party data, please ensure that the statement adheres to our policy

All the databases/datasets used in the study along with appropriately accessible links/accession-codes in the manuscript under the “Data availability” section as well as in this reporting summary. The raw RNA-seq and genotype data of the GTEx cohort are available to authorized users through dbGaP release, under accession

March 2021

code phs000424.v8.p2 [https://www.ncbi.nlm.nih.gov/projects/gap/cgi-bin/study.cgi?study_id=phs000424.v8.p2]. The raw RNA-seq and genotype of the ROS/MAP cohort are available in the AD Knowledge Portal under accession code syn3219045 [https://www.synapse.org/#!Synapse:syn3219045]. To access the ROS/MAP cohort, users need to complete and submit a data use certificate at https://adknowledgeportal.synapse.org/Data%20Access. The raw RNA-seq and genotype data of the PsychENCODE cohort are available in the PsychENCODE Knowledge Portal under accession code syn4921369 [https://www.synapse.org/#!Synapse:syn4921369]. Access to the PychENCODE cohort can be obtained by applying for access at https://www.nimhgenetics.org/request-access/how-to-request-access. The processed 3'aQTL summary statistics, 3'aTWAS models and all significant 3'aTWAS genes in 11 brain disorders are available at https://wlcb.oit.uci.edu/3aTWAS. Source data for uncropped blots are provided with this paper.

Human research participants

Policy information about studies involving human research participants and Sex and Gender in Research.

Reporting on sex and gender	Sex of participants was determined by GTEx, ROS/MAP and PsychENCODE cohorts. The sex determination methods can be found in the cited manuscripts. We downloaded these sex information from these cohorts and use sex as a covariate in QTL and TWAS analyses.
Population characteristics	GTEx cohort contained individuals with genotype and gene expression measured across 54 tissues. Further information regarding samples can be found in the cited manuscripts. ROS/MAP cohort contained individuals with genotype and gene expression measured in DLPFC. Further information regarding samples can be found in the cited manuscripts. PsychENCODE cohort contained individuals with genotype and gene expression measured in DLPFC. Further information regarding samples can be found in the cited manuscripts.
Recruitment	We did not recruit study participants.
Ethics oversight	Participants' data is de-identified and did not qualify as Human Subjects Research.

Note that full information on the approval of the study protocol must also be provided in the manuscript.

Field-specific reporting

Please select the one below that is the best fit for your research. If you are not sure, read the appropriate sections before making your selection.

☒ Life sciences ☐ Behavioural & social sciences ☐ Ecological, evolutionary & environmental sciences

For a reference copy of the document with all sections, see nature.com/documents/nr-reporting-summary-flat.pdf

Life sciences study design

All studies must disclose on these points even when the disclosure is negative.

Sample size	Sample size was determined based on the availability of existing GTEx, ROS/MAP and PsychENCODE data.
Data exclusions	We excluded RNA-seq samples without matched WGS data. When multiple samples were derived from the the same individuals, we selected the samples with the highest RNA integrity numbers (RIN).
Replication	The experiments has been performed independently with biological triplicates.
Randomization	The samples have been assigned randomly at the beginning of experiments.
Blinding	The bioinformatics analyses have been corroborated with blinded wet lab experiments.

Reporting for specific materials, systems and methods

We require information from authors about some types of materials, experimental systems and methods used in many studies. Here, indicate whether each material, system or method listed is relevant to your study. If you are not sure if a list item applies to your research, read the appropriate section before selecting a response.

Materials & experimental systems

n/a	Involved in the study
☐	☒ Antibodies
☐	☒ Eukaryotic cell lines
☒	☐ Palaeontology and archaeology
☒	☐ Animals and other organisms
☒	☐ Clinical data
☒	☐ Dual use research of concern

Methods

n/a	Involved in the study
☒	☐ ChIP-seq
☒	☐ Flow cytometry
☒	☐ MRI-based neuroimaging

Antibodies

Antibodies used

TDP-43 (Proteintech, 12892-1-AP, 1:3000), phospho-TDP-43 S409/S410 (Proteintech, 22309-1-AP, 1:3000), ATXN3 (Millipore-Sigma, MAB5360, clone 1H9, 1:2000), alpha-tubulin (ThermoFisher, 62204, clone DM1A, 1:3000)
The Secondary antibodies used in the study were ThermoFisher, A16078 [goat anti-mouse HRP, 1:3000], A16110 [goat anti-rabbit HRP, 1:3000], A32728 [goat anti-mouse Alexa FluorTM 647, 1:1000], A32733 [goat anti-rabbit Alexa FluorTM 647, 1:1000])

Validation

TDP-43 (Proteintech, 12892-1-AP): 12892-1-AP targets TDP-43 (C-terminal) in WB, IP, IHC, IF, CoIP, chIP applications and shows reactivity with human, mouse, rat samples. References are available here: https://www.ptglab.com/products/TARDBP-Antibody-12892-1-AP.htm

Phospho-TDP-43 S409/S410 (Proteintech, 22309-1-AP): 22309-1-AP targets Phospho-TDP43 (Ser409/410) in WB, IHC, IF applications and shows reactivity with human, mouse samples. References are available here: https://www.ptglab.com/products/phospho-409-410--TDP43-Antibody-22309-1-AP.htm

ATXN3 (Millipore-Sigma, MAB5360): Ataxin-3. The epitope was mapped precisely at E214-L233. MAB5360 can be used to study wild type ataxin-3 and the mutant form with polyglutamine expansion found in patients affected with spinocerebellar ataxin type 3/ Machado-Joseph disease (SCA3/MJD). In analysis of human tissues by Western blot, MAB5360 releaved several isoforms of ataxin-3 (presumably generated by alternative splicing, Trottier et al. 1998). The antibody detected polyglutamine aggregate (or nuclear inclusions) by IHC on SCA-3/MJD brain sections (Paulson et al. 1997). Detect Spinocerebellar Ataxia Type 3 using this Anti-Spinocerebellar Ataxia Type 3 Antibody, clone 1H9 validated for use in ELISA, IC, IH, IP & WB. References are available here: https://www.emdmillipore.com/US/en/product/Anti-Spinocerebellar-Ataxia-Type-3-Antibody-clone-1H9,MM_NF-MAB5360#anchor_REF

alpha-tubulin (ThermoFisher, 62204): 62204 detects alpha tubulin in human, mouse, rat, bovine, porcine, chicken, amphibian, gerbil and guinea pig samples.62204 has been successfully used in Western blot, immunohistochemistry, immunoprecipitation, electron microscopy, immunocytochemistry and immunofluorescent applications. The 62204 immunogen is native chick brain microtubules. This antibody recognizes an epitope within amino acids 426-450 of alpha tubulin. References are available here: https://www.thermofisher.com/antibody/product/alpha-Tubulin-Antibody-clone-DM1A-Monoclonal/62204

Eukaryotic cell lines

Policy information about cell lines and Sex and Gender in Research

Cell line source(s)

The HEK293T and SH-SY5Y cell lines were purchased from ATCC.

Authentication

The HEK293T and SH-SY5Y cell lines were authenticated by STR profiling by ATCC.

Mycoplasma contamination

The HEK293T and SH-SY5Y cell lines tested negative for mycoplasma with the MycoAlert Plus Mycoplasma Detection Kit (Lonza, LT07-710)

Commonly misidentified lines
(See ICLAC register)

No commonly misidentified cell lines were used in this study.
